# Ensuring vaccine access through local production: the need for a harmonized and sustainable approach

**DOI:** 10.1093/jlb/lsaf020

**Published:** 2025-10-31

**Authors:** Chimdessa Tsega

**Affiliations:** Faculty of Law, Chinese University of Hong Kong, Sha Tin, Hong Kong SAR

**Keywords:** vaccine access, local production, harmonization, sustainability, China-Africa

## Abstract

Local vaccine and pharmaceutical production has transitioned from a mere policy option to an essential requirement in the aftermath of the COVID-19 pandemic. Developed countries' vaccine nationalism and hoarding through bilateral agreements with manufacturers left African countries with minimal access to vaccines. As Africa strives for self-sufficiency in vaccine development and pharmaceutical manufacturing by aiming to produce 60 percent of the continent’s vaccine doses through indigenous manufacturing by 2040, it is crucial to examine existing initiatives and the roles of foreign players, especially China. In the first section, this article examines and evaluates regional initiatives aimed at promoting local production in Africa, focusing on efforts at global, continental, and regional levels to boost the manufacturing of vaccines and pharmaceuticals on the continent. The second section shifts focus to China’s role in Africa’s healthcare sector and highlights Chinese-backed pharmaceutical companies involved in vaccine and pharmaceutical production on the continent. The paper concludes by advocating for a harmonized and sustainable approach to local manufacturing. Specifically, the article argues that for these initiatives, whether government-backed or private investment, to succeed, there is a critical need to harmonize regulations, streamline procurement, diversify production, and address the challenge posed by intellectual property rights.

## I. LOCAL VACCINE AND PHARMACEUTICAL PRODUCTION IN A POST-COVID-19 WORLD: INTRODUCTION

The development of local vaccine and pharmaceutical production in low- and middle-income countries was not considered a priority before the pandemic. Instead, the emphasis was placed on ensuring access to affordable medical products, primarily achieved through the reduction of drug prices as Indian and subsequently Chinese generic drug manufacturers improved availability.^1^ As a result, it was economically unfeasible to invest in local production to compete with these generic suppliers. Moreover, several challenges inhibited local production efforts, including inadequate infrastructure, market failures, and regulatory hurdles.^2^ Many countries faced difficulties in establishing the necessary facilities and supply chains to support pharmaceutical manufacturing. Regulatory environments were often complex. Also, a shortage of skilled personnel in the pharmaceutical industry further hindered the ability to develop and maintain competitive local manufacturing capabilities.^3^

The pandemic has triggered a re-evaluation of policies surrounding local vaccines and pharmaceutical production. First, the critical need for domestic production of medical products such as ventilators, masks, and diagnostic tools became evident as shortages plagued healthcare across the globe. This underscored the necessity of having robust local manufacturing capabilities to ensure timely access to medical products.^4^ Second, the inequitable distribution of vaccines revealed the importance of local production. While developed countries successfully procured COVID-19 vaccines through partnerships with major vaccine suppliers, low- and middle-income countries often faced delays and limited access.^5^ Even though 11 billion doses of COVID-19 vaccines were produced in 2021, arguably sufficient for global immunization, vaccine hoarding and supply chain challenges made the distribution inequitable.^6^ Third, the pandemic also exposed significant vulnerabilities in global supply chains, as widespread shortage of essential medications and medical supplies showed the risk of relying on foreign manufacturers; countries must develop self-sufficient manufacturing capabilities to mitigate risks associated with global disruptions.^7^ Lastly, recent geopolitical events such as the conflict in Ukraine has disrupted global pharmaceutical supply chain by increasing logistical costs and restricting access to raw materials, revealing fragilities in international trade.^8^ These critical insights have prompted a transformative shift of local production policies.

It can also be argued that the embrace of local production is also closely linked to the resurgence of industrial policy, which aims to enhance local manufacturing capabilities to maintain innovative supremacy and reduce dependence on foreign suppliers through active state involvement.^9^ This strategic shift in economic policy is particularly relevant to the health sector, as the pandemic has highlighted the risks associated with relying on foreign suppliers and market driven responses to public health emergencies. Instead, there is a pressing need for targeted government interventions that focus on building and enhancing local manufacturing capabilities and ensure robust supply chains.^10^

Studies indicate that countries with robust local manufacturing capabilities tend to achieve higher vaccination coverage during the COVID-19 pandemic compared to those who rely solely on imports.^11^ This correlation suggests that investments in domestic production not only enhance access to vaccines but also improve overall public health outcomes. According to WHO, one in five children in Africa does not receive all the necessary and basic vaccines needed to protect them from preventable diseases.^12^ As a result, >30 million children under 5 across the continent suffer from vaccine-preventable diseases. Tragically, this leads to the death of over half a million children annually. This highlights the urgent need to improve vaccine access and healthcare infrastructure in the region.^13^

All African countries import the majority of their pharmaceutical and medical needs. Specifically, Africa imports >70 percent of its total medicines, >90 percent of its medical devices, and >99 percent of its vaccines.^14^ The COVID-19 pandemic has highlighted significant challenges in Africa’s vaccine production and distribution. Africa only accounts for 3 percent of the global production of medical products and 0.1 percent of global vaccine production.^15^ Specifically with respect to COVID-19 vaccines, as of September 2021, Africa manufactured only <1 percent of all the COVID-19 vaccines administered on the continent.^16^ The continent heavily relied on imports and donations, with more than half of the vaccines (53 percent) obtained through donation.^17^

Investments in local vaccine and pharmaceutical manufacturing enhances access as they create stable and reliable supply chain. Local production also decreases costs associated with procurement, allows for quicker responses to outbreaks, and enables tailoring production to local epidemiological needs. Despite the critical importance of vaccine research in enhancing production capabilities and tailoring vaccine development to the specific needs of the region, research and development (R&D) in the sector remains minimal.^18^ Currently, local production on the continent is predominantly focused on generic medicines with simple processes, which accounts for 70 percent of the total local pharmaceutical production.^19^ This production focuses on downstream processes such as packaging, and filling and finishing activities, with minimal emphasis on upstream R&D or the limited production of intermediates and active pharmaceutical ingredients (APIs), which require complex chemical and biological processes.^20^

Furthermore, the concentration of production is striking, with 80 percent of the continent’s 600 packaged medicine producers located in just eight countries, predominantly in North Africa.^21^ Ten countries account for 75 percent of the total market share.^22^ This uneven distribution further highlights the need for a diversified local manufacturing ecosystem on the continent.

By 2030, the African Development Bank (AfDB) estimates that an investment of 111 billion US dollars is needed across the continent, both through public and private sector interventions. Out of this, 11 billion US dollars should be allocated to the pharmaceutical industry, including the creation of R&D capabilities and the implementation of quality standards.^23^ Investing in local production is imperative, especially considering Africa’s experiences during the pandemic and the challenges of ensuring vaccinations and access to relevant medical products across the continent. During the pandemic, Africa experienced severe disruptions to healthcare systems, with many countries facing shortages of medical supplies and vaccines.^24^ As a result, Africa began to prioritize initiatives in local manufacturing and innovation to strengthen essential vaccine and pharmaceutical supplies. The next section surveys initiatives across the continent that are established toward such realization.

## II. CONTINENTAL INITIATIVES ON LOCAL VACCINES AND PHARMACEUTICAL MANUFACTURING

Local manufacturing of vaccines and other medical products has become a critical priority for the African continent in recent years. The region has faced significant challenges from outbreaks of diseases such as HIV/AIDS, Ebola, and COVID-19, which have disproportionately affected its populations. This vulnerability has largely stemmed from the continent’s heavy reliance on imported medical products.^25^ In response, various initiatives have been launched by the African Union and other regional organizations aimed at enhancing local manufacturing capabilities. These initiatives aim to reduce dependence on foreign imports, enhance self-sufficiency, and ensure that essential medical supplies are readily available during emergencies. The following section will assess these initiatives, analyzing their objectives, responsibilities, and contributions to improving local manufacturing of vaccines and other medical products. In addition, this discussion will include similar initiatives undertaken by global organizations, such as the UN and WHO, to support Africa’s local manufacturing capacity.

### II.A. African Union’s Pharmaceutical Manufacturing Plan for Africa

In May 2007, the African Union (AU) adopted the Pharmaceutical Manufacturing Plan for Africa (PMPA), a strategic initiative aimed at promoting the local production of pharmaceutical products.^26^ The goal is to increase access to affordable, high-quality medicines and ensure a sustainable supply of essential drugs. The plan emphasizes the importance of technology transfer, infrastructure enhancement, and building partnerships to support local manufacturing. The plan indicates that in addition to ensuring a stable supply of quality, affordable drugs, local manufacturing will also save foreign exchange, create jobs, facilitate technology transfer, and stimulate exports.^27^ Under the PMPA, further actions such as establishing list of priority essential medicines to be manufactured, establishing criteria on the eligibility of manufacturers, and identifying and assigning countries or a group of countries in relation to which medical product they produce are anticipated.^28^

In 2012, a detailed business plan was developed by the AU and African Union Development Agency (AUDA-NEPAD) to outline the approaches to strengthen the continent’s ability to produce high-quality, affordable medical products, which will improve health outcomes and boost industrial and economic growth. This document also highlighted several challenges, including the need for technical expertise, financial resources, and improved infrastructure.^29^ The plan emphasizes the AU’s focus on developing human capital, improving skills in the pharmaceutical industry, and strengthening industry partnerships to address these challenges.^30^

In light of this, AUDA-NEPAD published a report identifying the list of 24 priority medical products and a roadmap for regional manufacturing in January 2025.^31^ The report, aimed at scaling up local production of essential medicines, vaccines, and other health products, provides guidelines at regional and national levels on the manufacture of priority medical products on the continent. The list of these priority medical products is identified based on disease burden–relevant market and need factors such as manufacturing capacity, absence of intellectual property (IP) barriers, availability of technology transfer, raw materials, and regulatory capability.^32^ The list of priority medical products is categorized into six depending on need and market: high needs-high market, growing need-high market, low need-high market, high need-low market, growing need-low market, and low need-low market.^33^

The PMPA includes a roadmap with the goal of meeting at least 50 percent of the continent’s pharmaceutical needs by 2030. This roadmap is categorized into seven objectives with cross-cutting aims.^34^ The first objective is to secure political will and support for the PMPA by establishing platforms and strategic partnerships, to be led by the PMPA secretariat by 2025. The second objective focuses on promoting the implementation of a list of 24 priority medical products by harmonizing policies for local manufacturing across the continent, strengthening the continent’s IP framework to boost innovation, and streamlining the approval process for local production.

The third objective involves strengthening the workforce for local manufacturing through the establishment of Regional Centres of Excellence and the development of relevant national, regional, and continental platforms for medical products. Objective 4 focuses on strengthening the continent’s R&D and facilitating technology transfer for local manufacturing. This includes establishing five regional centers for collaborative research, facilitating technology transfer partnerships between African and global manufacturers, and strengthening regulatory systems.^35^

Enhancing the investment climate is the fifth objective, achieved through the harmonization of incentive programs, public–private partnerships, and improvements to the legal, regulatory, and business environment. The sixth objective is regulatory harmonization, specifically through capacity-building programs for national medicine regulatory authorities (NMRAs). The seventh and final objective is to improve supply chains and market access for the list of priority medical products through regional market data collection and pooled procurement in collaboration with RECs.

The PMPA’s focus on local pharmaceutical production and ensuring self-sufficiency in the sector is crucial in mitigating vulnerabilities exposed during global health crises, such as the COVID-19 pandemic, where supply chain disruptions underscored the risks of dependency on external sources. Moreover, the categorization of medical products based on market needs is a move that aligns production with actual healthcare demands, potentially optimizing resource allocation. Nonetheless, without adequate political commitment and systemic challenges such as the need for robust regulatory frameworks, lack of investment in infrastructure, and limited access to technology, the ambitious goal of meeting 50 percent of the continent’s pharmaceutical needs by 2030 may remain aspirational.^36^

### II.B. Africa CDC’s Partnerships for African Vaccine Manufacturing

In 2021, the AU established the Partnerships for African Vaccine Manufacturing (PAVM) in response to the challenges exposed by the COVID-19 pandemic. Supervised by the African Centre for Disease Control (AfCDC), PAVM aims to strengthen the African vaccine manufacturing sector with the ambitious goal of enabling the continent to develop, produce, and supply >60 percent of its required vaccine doses by 2040.^37^

Under this initiative, Africa plans to produce 1.5 billion vaccine doses annually by 2040, focusing on 22 diseases deemed critical across the continent.^38^ These diseases are categorized into three groups: legacy diseases, which are prevalent and offer economies of scale (such as tuberculosis, hepatitis B, and measles); expanding diseases, which either lack commoditized vaccines or have relatively high-priced options (such as HIV, malaria, and COVID-19); and outbreak diseases such as Ebola.^39^ To achieve these objectives, the PAVM framework for action outlines both the establishment of new plants and expansion of existing facilities. This plan envisions the development of 23 manufacturing plants, comprising 12 integrated drug substance and fill-and-finish plants, alongside 11 dedicated solely to fill-and-finish operations.^40^

PAVM advocates for various strategies to achieve this objective, specifically the creation of an African vaccine procurement pooling mechanism, establishing a vaccine manufacturing deal preparation and financing facility, strengthening national regulatory agencies and regional centers of regulatory excellence, facilitating transfer of vaccine technology and IP, creating regional capability and capacity centers, establishing vaccine R&D centers, advocacy for enabling trade policies for vaccines, and ensuring an effective continental strategy for delivery and oversight.^41^

In February 2024, the AU Assembly directed the AfCDC to upgrade PAVM to Platform for Harmonized African Health Manufacturing.^42^ This expansion broadens PAVM’s mandate from focusing solely on vaccines to encompassing the manufacturing of all health products, including therapeutics and diagnostics. Furthermore, the Assembly established the African Pooled Procurement Mechanisms (APPM), designed to streamline the procurement of medical products across the continent.^43^

In June 2024, a financing mechanism of 1.2 billion US dollars for the next 10 years was launched to accelerate the expansion of commercially viable vaccine production on the continent. As of October 2024, five manufacturers on the continent are set to produce eight vaccines by 2030 while additional five commercial-scale production facilities are awaiting technology transfer.^44^

**Table 1:** Existing and planned vaccine manufacturers in Africa

**Existing vaccine manufacturers**		**Planned manufacturers**	
**Drug substance**	**Fill and finish**	**Drug substance**	**Fill and finish**
▪ IP de Algerie (Algeria) ▪ Institut Pateur de Tunis (Tunisia) ▪ Ethiopian Public Health Institute (Ethiopia) ▪ Institut Pateur de Dakar (Senegal) ▪ Biovac (South Africa)	▪ Saidal (Algeria) ▪ Vascera (Egypt) ▪ Institut Pateur du Maroc (Morocco) ▪ Sothema (Morocco) ▪ Aspen Pharmacare (South Africa)	▪ Biovax (Kenya) ▪ Rwanda Biomedical Centre (Rwanda) ▪ BionTech (Rwanda) ▪ Innovative Biotech (Nigeria) ▪ NantBotswana (Botswana) ▪ NantSA (South Africa) ▪ DEI Biopharma (Uganda) ▪ Marbio (Morocco)	▪ Minapharm (Egypt) ▪ Pharco (Egypt) ▪ Eva Pharm (Egypt) ▪ Biogeneric (Egypt) ▪ Sensyo Pharmatec (Morocco) ▪ Galencia (Morocco) ▪ Recipharm (Morocco) ▪ Atlantic Biotech (Ghana) ▪ DEK (Ghana) ▪ Biovaccines (Nigeria) ▪ NIBI (Nigeria) ▪ Shieldvax (Ethiopia)

Source: Compiled by author from various sources.

A recent study on Africa’s capacity to produce antigens reveals that production levels remain significantly low, falling short of the PAVM’s domestic production targets.^45^ Existing manufacturers face not only a lack of support and inadequate technology transfer mechanisms but also limited commercial success, which threatens their sustainability. While PAVM has established ambitious goals, substantial efforts are still required to ensure long-term investment, create a supportive regulatory environment, foster commercial viability, and implement diverse technology transfer initiatives.

### II.C. Africa Development Bank’s African Pharmaceutical Technology Foundation

Amid the challenges brought about by the COVID-19 pandemic, Africa’s struggle to access essential vaccines and diagnostics prompted AfDB to launch the African Pharmaceutical Technology Foundation (APTF).^46^ The goal is to enhance access to and use of technologies in Africa’s pharmaceutical sector by creating new pathways for R&D, production, certification, and deployment of new drugs, vaccines, and diagnostics.^47^ The focus is on diseases that are widely prevalent on the continent, including current and future pandemics. One of the key responsibilities of the APTF is to promote alliances between foreign and African pharmaceutical companies and strengthening local companies to engage in local production initiatives. However, it is yet to be seen how the APTF is planning to undertake such engagement.^48^

At a recent conference, the Deputy Chairperson of the AUC indicated that the APTF will enhance partnerships between global and African companies by supporting the commercialization of IPRs by African companies and its use for technology transfer, through licensing, sharing of tacit know-how, and by building domestic production capacity.^49^ Among the mandate of the APTF is identifying and advocating for the use of IP flexibilities. The foundation emphasizes the importance of training and capacity building on TRIPS enforcement and leveraging flexibilities to stimulate innovation in the pharmaceutical sector.^50^ For instance, one significant advantage of TRIPS flexibilities is the provision for compulsory licensing, which allows countries to produce or import generic versions of patented medicines without the consent of the patent holder, particularly in public health emergencies.^51^ This mechanism can be vital for African nations facing health crises, such as pandemics, as it enables them to access vaccines at reduced costs and enhance local manufacturing capabilities. By utilizing compulsory licensing, the APTF can advocate for policies that support local pharmaceutical companies. However, there are limitations to TRIPS flexibilities that the APTF must navigate carefully. First, the process of obtaining a compulsory license can be lengthy and bureaucratically complex, often deterring local manufacturers from pursuing this option.^52^ Additionally, the political and economic pressures from developed countries, which may oppose the use of such flexibilities, can create an environment of uncertainty that hinders local production efforts.^53^

Moreover, the APTF seeks to establish regional centers of excellence in pharmaceutical production through initiatives like university–industry collaboration. However, the APTF must outline specific tasks that it intends to undertake to foster this partnership, such as potential international partners, areas of vaccines, and incentive mechanisms.

### II.D. AUDA-NEPAD’s Africa Medicines Agency

The treaty establishing the Africa Medicines Agency (AMA) was adopted in February 2019 and came into force in November 2021 following the ratification of the treaty by 15 AU members.^54^ The AMA treaty is currently ratified by 19 African states.^55^ AMA is a specialized agency of the AU established with a mandate of regulating medical products on the continent.^56^ AMA’s main objective is harmonizing regulatory frameworks of medical products and coordinating the collection, management, storage, and sharing of information on all medical products among state parties and globally.^57^ This is critical because regulatory challenges such as weak legislative frameworks, slow and fragmented registration process for medicines, long approval process, and limited technical capabilities are among the major challenges of local manufacturing on the continent.^58^

The agency is part of the broader goal of bolstering local pharmaceutical production on the continent as part of the PMPA and support trade under the AfCFTA. By aligning technical requirements for developing and marketing medicines across the continent, the AMA aims to build a unified regulatory framework and enhance regulatory competence.^59^ This is specifically carried out through close collaboration with National Medicines Regulatory Authorities to identify standard and falsified medical products and facilitate information sharing among member countries. In addition, AMA is conferred with the responsibility to oversee joint reviews clinical trials applications and coordinate inspections of manufacturing sites for APIs.^60^ This streamlined regulatory approach is meant to enhance the continent’s capacity to respond to health emergencies and ensure proper access to medical products and technologies.

### II.E. UN Economic Commission for Africa’s AfCFTA-Anchored Pharma Initiative

In November 2019, the AfCFTA-Anchored Pharmaceutical Initiative (Pharma Initiative) was launched by the United Nations Economic Commission for Africa to leverage the AfCFTA and AMA to improve access to essential medical products on the continent.^61^ Pharma Initiative is launched with the aim of ensuring sustainable access to equitable, safe, and affordable quality medicines, and create fiscal space for African countries by optimizing pharmaceutical procurement and production.^62^ The Initiative has a three-pronged approach to attaining these objectives: facilitation and advocacy for local pharmaceutical production, ensuring harmonized regulatory and quality standards of medicines, and centralized pooled procurement of pharmaceuticals.^63^ A Centralized Pooled Procurement Mechanism (CPPM) was officially launched in July 2021.^64^

Under the Pharma Initiative, 10 priority Sexual, Reproductive, Maternal, Neonatal & Child Health (SRMNCH) products have been selected for pooled procurement in 10 pilot countries: Comoros, Djibouti, Eritrea, Ethiopia, Kenya, Madagascar, Mauritius, Rwanda, Seychelles, and Sudan. The initiative is structured into three phases: Phase I (2019–20), Phase II (2021–22), and Phase III (2023–24).^65^ Under Phase I, the Initiative focused on conducting situational analysis and developing frameworks leading to the validation and adoption of the CCPM in the 10 countries. Under Phase II, the Initiative published GMP for pharmaceutical manufacturing and regulatory affairs in the 10 pilot countries.^66^ Phase III focused on developing the CCPM and its legal framework including the prioritization of the 10 SRMNCH products. Under Phase III, a draft agreement establishing the CCPM is developed and expected to be signed by member states.^67^

While promoting local manufacturing remains a core objective of the Pharma Initiative, the specific tasks it plans to undertake are yet to be clearly defined. Currently, the Initiative’s mandate is centered on ‘facilitation and advocacy.’ Nevertheless, its focus on harmonizing regulatory affairs and Good Manufacturing Practices (GMP) is a critical step toward strengthening local production. In this context, the launch of the CCPM will play a pivotal role in securing demand for both existing and emerging manufacturers across the continent. This, in turn, will support the growth and sustainability of local pharmaceutical production.

### II.F. The East African Community’s Pharmaceutical Manufacturing Plan of Action

The Eat African Community (EAC) adopted the Pharmaceutical Manufacturing Plan of Action (EAC-RPMPOA) as a strategic roadmap toward fostering a strong regional pharmaceutical manufacturing industry.^68^ Fist launched in 2012, the EAC-RPMPOA (2012–16) aims to establish a robust local pharmaceutical industry that can meet the health needs of the population by producing safe, effective, and quality medicines. This plan outlines different targets including regulatory harmonization, public–private partnership, regulatory capacity building, and boosting innovation through R&D.^69^ Accordingly, the plan prioritizes initiatives such as harmonizing policies across national and regional levels and integrating public health–related WTO TRIPS flexibilities into the legal frameworks of partner states.^70^ However, despite these ambitious goals, the implementation of the plan has faced significant challenges, primarily due to its reliance on the commitment of state parties and the scarcity of resources necessary for effective implementation.^71^

In August 2017, a revised version of the EAC-RPMPOA was adopted, outlining a comprehensive roadmap to strengthen the region’s pharmaceutical manufacturing capabilities from 2017 to 2027.^72^ The plan iterated the need to reduce pharmaceutical imports from outside the EAC countries to <50 percent. It specifically mandated that at least 50 percent of procurement of medicines by national agencies should be sourced from local producers within the EAC region.^73^ To facilitate its effective implementation of the plant within EAC states, a steering committee is established at the EAC level to closely work with national focal points.

Furthermore, the plan encourages EAC countries to capitalize on the extension granted by the WTO to Least Developed Countries (LDCs), allowing them to utilize health-related TRIPS flexibilities until 2033. With six out of eight EAC countries classified as LDCs, there is a pressing need for more concrete measures to domesticate and consolidate these flexibilities.^74^ In this context, the plan underscores that the lack of approximation of national laws by the partner states to take advantages of available flexibilities continues to pose a significant challenge in the region.^75^ In 2013, the EAC adopted a policy on TRIPS flexibilities, which was subsequently followed by the EAC Protocol on TRIPS flexibilities, which remains unratified.^76^ Studies indicate that, despite these policies and the EAC-RPMPOA, TRIPS flexibilities are largely underutilized and often unknown to pharmaceutical producers in the region.^77^ One notable achievement of the first EAC-RPMPOA is regulatory harmonization. Launched in March 2012 under the support of the African Medicines Regulatory Harmonization Initiative (AMRH), this initiative aimed to create a standardized medicines registration process across member countries.^78^ By utilizing common documents, processes, and shared information, it has successfully ensured Good Manufacturing Practices (GMPs) and facilitated cost and time savings through a streamlined marketing authorization process in member states.^79^ The EAC-RPMPOA 2017–2027 plan also aims to stimulate increased investment in pharmaceutical production by introducing an EAC model for national incentive packages, encompassing tax incentives, preferential pricing, support in public procurement, and streamlined import classification.^80^

### II.G. Economic Community of West African State’s Regional Pharmaceutical Plan

In April 2014, the West African Health Organization (WAHO) adopted the ECOWAS Regional Pharmaceutical Plan (ERPP), officially adopted by the Assembly of Health Ministers.^81^ The plan aims to enhance local pharmaceutical production in member states that can produce and supply safe and effective high-quality medicines for local, regional, and international markets.^82^ A key objective of the ERPP is to reduce reliance on pharmaceutical imports by increasing local production from 30 percent to 60 percent, and support local industry development to meet regional needs and become a source of high-quality exports.^83^ The ERPP also supports local clinical trials and strengthens medicine regulatory harmonization processes. By promoting collaboration and harmonizing regulations, the ERPP aims to overcome obstacles such as disparate medicine registration processes and varying patient access within the ECOWAS region.^84^

The ERPP identifies significant challenges in local drug manufacturing, particularly antiretrovirals, encompassing issues such as low regional manufacturing capacity, the absence of a harmonized regulatory framework, and discrepancies between demand and supply.^85^ The ERPP Resolution underscored the imperative to enhance local pharmaceutical manufacturing, urging member states to create a conducive environment for this purpose.^86^

As part of the ERPP implementation and recognizing the importance of good medical practices for safety and effectiveness of medical products, WAHO published a regional roadmap on good manufacturing practices for pharmaceutical manufacturing in the region.^87^

### II.H. WHO’s mRNA Vaccine Technology Transfer Hub

During the COVID-19 pandemic, inequities in vaccine distribution and disruptions to the global medical supply chain prompted the WHO to initiate the mRNA Vaccine Technology Transfer Hub in June 2021.^88^ Based in Cape Town, South Africa, the hub aims to build mRNA vaccine production capacity in low- and middle-income countries (LMICs).^89^ WHO selected Afrigen Biologics, a South African company, to establish the mRNA vaccine production technology, with support from the South African Medical Research Council (SAMRC), which is mandated to provide research, and Biovac, a South African vaccine company tasked with being the first vaccine manufacturing bespoke.^90^ Within this consortium, SAMRC is coordinating the regional R&D program. The hub provides technology development, training, and technology transfer for a network of technology recipients (partners) in LMICs, which will then produce and sell vaccines commercially.^91^ The Medicines Patent Pool (MPP), a WHO backed organization based in Geneva, is in charge of the fundraising and legal needs for the hub, including support on intellectual property matters.^92^ The program is guided by three key principles, which are equitable access to mRNA technologies, creating value and share intellectual property through open access innovation, and promote sustainable capacity to produce mRNA vaccines with coherent policies and adequate investments.^93^

As of February 2025, 15 partners are selected as recipients of technology transfer, 13 of which have signed a transfer technology agreement to receive technology transfer, out of which 5 signatories are based in Africa.^94^ The implementation cycle of the technology transfer takes four major cycles: developing the mRNA technology through research and development, developing human capital through training, mRNA technology transfer to partners, and finally establishing the mRNA vaccine technology in LMICs.^95^

Even though the primary focus of the hub was COVID-19 vaccines, the hub is designed to encourage the development of other mRNA vaccines and therapeutics against most prevalent diseases in LMICs.^96^ Nine disease areas in Africa are identified as primary targets: Crimean-Congo hemorrhagic fever, COVID-19, HIV, leishmaniasis, malaria, rabies, respiratory syncytial virus (RSV), Rift Valley fever (RVF), and tuberculosis (TB).^97^ The development of five mRNA vaccine products, specifically mRNA vaccines for RSV, RVF, gonorrhea, HIV, and TB, has already commenced.^98^

### II.I. GAVI’s African Vaccine Manufacturing Accelerator

GAVI is a public–private partnership launched in 1999 in partnership with WHO, UNICEF, The Bill and Melinda Gates Foundation, the World Bank, CSOs, vaccine manufacturers, and other partners with the objective of protecting health through the equitable and sustainable use of vaccines.^99^ In June 2024, GAVI launched the African Vaccine Manufacturing Accelerator (AVMA), a financing initiative designed to provide >1 billion US dollars in financial incentives to vaccine manufacturers in Africa.^100^ According to the GAVI’s guideline for the manufacturers, the AVMA has two objectives: creating a sustainable manufacturing base that contributes to healthy global vaccine markets and improved African pandemic and outbreak vaccine supply resilience.^101^

AVMA supports African vaccine manufacturers involved in either a full-scale production or fill and finish operations. The vaccines must be those within GAVI’s portfolio and among the list of priority vaccines established by the AVMA. These vaccines include Oral Cholera Vaccine, Malaria, Measles-Rubella, Hexavalent, Yellow Fever, Ebola, Rotavirus, and Pneumococcal, and AVMA priority technology platforms, which include mRNA and Viral Vector.^102^ Local manufacturers in Africa have two options to obtain payments from the AVMA: milestone payment and accelerator payment.^103^ Milestone payment is applicable to those engaged in the production of either AVMA vaccine priorities or AVMA priority technology platforms. However, the manufacturer must have received WHO prequalification following WHO’s evaluation that the vaccine is safe and effective and depending on whether the vaccine is among priority vaccines under WHO’s list of prequalified vaccine and supply security of the vaccine in question.^104^ The accelerator payment, on the other hand, is paid upon the delivery of vaccines subject to UNICEF tenders. This is a per-dose payment separate from the UNICEF tender contract with the manufacturer.^105^

**Figure 1:**
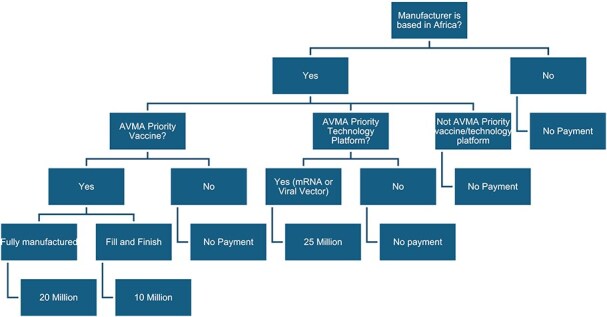
Visual representation of GAVI’s AVMA Financing Initiative.

In total, a manufacturer’s total disbursement as part of AVMA is capped at 250 million US dollars for manufacturing activities. For solely fill-and-finish operations, the cap is set at 50 million US dollars.

## III. THE GROWING ROLE OF CHINESE COMPANIES IN AFRICA’S VACCINE AND MEDICAL PRODUCT MANUFACTURING SECTOR

China is one of the largest producers and exporters of pharmaceuticals globally, with significant influence in Africa. Alongside India, China serves as a major supplier of medical products to the continent.^106^ As trade and investment relations between Africa and China continue to grow, the pharmaceutical sector presents an opportunity to enhance local manufacturing capabilities. By attracting Chinese companies to establish manufacturing facilities in Africa, partnering with local firms, and engaging in technology transfer initiatives, the continent can strengthen its pharmaceutical industry. The following section offers a brief analysis of the policy context and recent involvement of Chinese companies in African vaccine and pharmaceutical manufacturing.

### III.A. China-Africa Health Care Cooperation

The launch of the Forum on China-Africa Cooperation (FOCAC), held first in Beijing in 2000, marked a new chapter in the relationship.^107^ This multilateral framework has transformed China’s engagement with African countries thereby propelling the diplomatic and trade ties between the two. Over eight triennial summits, FOCAC has transformed from a development-focused initiative into a comprehensive multilateral framework addressing trade, security, infrastructure, and energy matters among others. The recent FOCAC Summit, which was attended by 51 African heads of states/governments, marked another milestone where China pledged a 50-billion-US-dollar financial support to Africa over the next 3 years (2024–27).^108^

This partnership is further strengthened under the Belt and Road Initiative (BRI), China’s global infrastructure development strategy launched in March 2013 and officially incorporated into the Constitution of the Communist Party in October 2017.^109^ A key component of the BRI is the Health Silk Road (HSR), which focuses on public health and international cooperation in healthcare.^110^ Formally proposed for the first time during President Xi Jinping’s visit to Uzbekistan in 2016, the Health Silk Road aims to enhance healthcare collaboration among BRI countries, with a Memorandum of Understanding signed with WHO the following year.^111^

A BRI document published in 2015 indicates that the health sector is an important axis of China–Africa cooperation.^112^ In addition to strengthening international collaboration on information sharing and healthcare technologies to address public health emergencies, the document notes China’s commitment in providing medical assistance and emergency medical aid to relevant countries under the BRI.^113^ In August 2017, China convened Belt and Road High Level Meeting for Health Cooperation, where representatives from the health sectors of >30 countries participated.^114^ Among the main objectives of the meeting was to strengthen cooperation in medical R&D, provide assistance along the BRI countries, and establish a health policy network and health-industry sustainable alliance.^115^

In addition, under the China-Africa Cooperation Vision 2035 plan—a document co-developed by China and Africa—both parties agreed to create synergy between the BRI and African development agenda as envisaged in Agenda 2063.^116^ China also affirmed that it will ‘help’ Africa develop its manufacturing sector and improve technical standard system for quality infrastructure. This includes cooperation and assistance in healthcare areas such as improving medicine accessibility and affordability, and medical research.^117^

According to the Beijing Action Plan (2025–27), a plan adopted following the ninth Ministerial Summit of the FOCAC, healthcare is one of the 10 partnership initiatives between China and Africa.^118^ In this regard, the plan outlines that both sides will host the Health Silk Road Cooperation Conference and China-Africa Ministerial Forum on Health Cooperation. In particular, China will support investing in Africa’s pharmaceutical and medical equipment industry through co-investments between Chinese and African private sectors. This includes encouraging Chinese private enterprises to invest in pharmaceutical sector in Africa.^119^

### III.B. COVID-19 and a New Height for the Health Silk Road in Africa

Following the outbreak of the COVID-19 pandemic, HSR became an integral part of China’s response and cooperation. In particular, in June 2020, 24 countries signed the ‘Joint Statement of the High-Level Video Conference on Belt and Road International Cooperation: Combating COVID-19 with Solidarity.’^120^ Particularly with African countries, China convened an extraordinary China Africa Summit on Solidarity Against COVID-19 via video link on June 17, 2020. At the end of the summit, which was attended by the Head of State or Government of 13 African countries and the Heads of both the UN and WHO, China affirmed its commitment to deliver initiatives and measures aimed at helping Africa to build capacity for disease prevention and control, including expediting the construction of Africa CDC headquarters.^121^ China’s medical aid to Africa usually takes one of the following five forms: dispatch of medical teams; building of hospitals and medical infrastructure; the donation of drugs and medical equipment; training of healthcare workers; and the control and prevention of infectious diseases, including COVID-19.^122^

According to the Chinese State Council, China has provided >2.2 billion doses of COVID-19 vaccines.^123^ Studies by international organizations and media groups also indicate that although the numbers slightly vary, China was the largest exporter of COVID-19 vaccines in 2021.^124^ Following the pandemic’s outbreak, China’s initial response included convening a meeting with African heads of state and governments to emphasize the need for solidarity and discuss a unified approach to combating the virus.^125^

In February 2021, China pledged to provide COVID-19 vaccines to 19 African countries.^126^ This was followed by a new pledge of 1 billion doses to Africa at the completion of the FOCAC ministerial meeting in November 2021, with 600 million doses provided as a donation and 400 million doses to be produced jointly by Chinese companies and relevant African countries.^127^ As part of this initiative, manufacturers in three African countries (Saidal of Algeria, Vacsera of Egypt, and Sotherma of Morocco) entered into a partnership agreement with Chinese companies and locally manufactured COVID-19 vaccines.^128^ Africa was the third largest recipient of Chinese COVID-19 vaccines, after Asia Pacific and Latin America.^129^

**Table 2:** Local COVID-19 vaccine manufacturers in Africa

**Local manufacturer**	**Country**	**Vaccine type**		**Manufacturing type**	**Annual manufacturing capacity**
Saidal	Algeria	Sinovac	2021	Fill & finish	96 million
Vacsera	Egypt	Sinovac	2021	Drug substance and fill & finish	100 million
Sotherma	Morocco	Sinopharm	2021	Drug substance	60 million

Source: Compiled by author from various sources.

Another key platform that China utilized to further its international healthcare cooperation was the COVAX. COVAX was a multilateral initiative led by GAVI, WHO, UNICEF, and the Coalition for Epidemic Preparedness Initiative to accelerate the development, manufacture, and fair and equitable distribution of COVID-19 vaccines until the end of 2023.^130^ China, though reluctant to joining the COVAX alliance at the beginning, was an active participant in the initiative’s vaccine manufacturing and distribution activities.^131^ In February 2021, China pledged to contribute 10 million vaccine doses to COVAX, and later pledged 100 million US dollars to enhance vaccine access for low-income countries.^132^ Including both donations and commercial sales, China has delivered ~232 million doses of COVID-19 vaccines (114 million doses of Sinopharm and 118 million doses of Sinovac) through the COVAX initiative as of late 2021.^133^

It is undeniable that the COVID-19 pandemic has significantly amplified the role and importance of the HSR. China’s multi-faceted approach, encompassing vaccine provision, medical aid, and active participation in global initiatives like COVAX, has positioned the HSR as a crucial instrument for international healthcare cooperation. While questions of efficacy and geopolitical motivations persist, the HSR has undeniably facilitated the distribution of vital resources during a global health crisis, particularly to African nations.

As African countries prioritize health infrastructure in their post-pandemic plans, it is critical to leverage the growing partnership with China through both FOCAC and HSR so as to enhance Chinese companies in setting up manufacturing bases in Africa either through direct investments by Chinese companies or in partnership with African companies.

### III.C. Chinese Companies Involved in the Manufacturing of Medical Products in Africa

Despite the increasing trade and investment ties between China and Africa, Chinese investment in Africa’s pharmaceutical manufacturing sector remains notably limited. Over the past two decades, China has emerged as one of Africa’s largest trading partners, with bilateral trade reaching over 254 billion US dollars in 2021, according to the China-Africa Research Initiative.^134^ Much of this investment, however, has been concentrated in infrastructure, natural resources, and energy sectors, leaving critical industries such as pharmaceutical manufacturing underdeveloped. While Chinese companies have demonstrated substantial capacity in pharmaceutical production domestically, this expertise has yet to be meaningfully leveraged to build sustainable manufacturing capabilities within African nations. Addressing this gap could not only strengthen Africa’s healthcare systems but also align with broader goals of industrialization and self-reliance under the African Union’s Agenda 2063.^135^

Following the latest FOCAC Summit in September 2024, China unveiled 10 partnership action plans, one of which focuses on public health and Africa’s pharmaceutical industry.^136^ The plan acknowledges China’s role in helping Africa address the COVID-19 pandemic, specifically the 240 million vaccine doses that China donated to 53 African countries plus medical expert teams dispatched to 17 African countries to fight the pandemic.^137^ The plan specifically highlights the commitment to supporting local pharmaceutical manufacturing in Africa noting that:

Both sides support promoting the development of pharmaceutical production and the medical equipment industry in Africa including access to active pharmaceutical ingredients (API) through co-investment by Chinese and African private sectors, so as to help Africa reduce medicine and medical equipment imports and safeguard medical sovereignty. China will continue to encourage and support Chinese private enterprises in investing in Africa’s pharmaceutical industry.^138^

At the Summit, President Xi also explicitly noted that China will support and encourage Chinese companies to invest in Africa’s pharmaceutical production.^139^ The HSR and FOCAC are critical instruments providing policy support to Chinese firms wanting to expand into the African market. By leveraging these frameworks, Chinese companies have lately strengthened their expansion into the African Pharmaceutical ecosystem.^140^ While some companies invest directly in establishing manufacturing facilities, contributing to the development of local production capacity, and reducing dependency on imports, others focus on forming partnerships with existing African companies (see table below). These partnerships facilitate technology transfer, knowledge sharing, and capacity building.^141^

For Chinese companies, investing in the African pharmaceutical market presents both opportunities and challenges. On one hand, Africa’s pharmaceutical market is growing rapidly, fueled by a rising population, urbanization, and increasing healthcare needs. According to Afrexim Bank, Africa’s pharmaceutical industry is projected to be worth 46 billion US dollars by 2030, driven by improved healthcare infrastructure and rising demand for medicines.^142^ This growth has been further accelerated by the COVID-19 pandemic. These trends make the African pharmaceutical sector an attractive investment opportunity for Chinese companies seeking to expand into emerging markets.^143^ However, the pharmaceutical industry is vastly different from low-capital sectors like textiles and clothing, which have traditionally attracted Chinese investment in Africa. Pharmaceutical manufacturing is a capital-intensive industry, requiring significant upfront investment in infrastructure, advanced technology, and R&D.^144^

**Table 3:** Chinese companies engaged in non–COVID-19 pharmaceutical production in Africa (2015–2025)

**Chinese company**	**Host African country**	**Medical product**	**Estimated capital (US$)**	**Year**
SanSheng Pharmaceuticals	Ethiopia	Tablets, capsule, injectables	85 million	2018
Shanghai Fosun Pharmaceutical	Ivory Coast	Antimalaria and antibiotics	55 million	2024
Jijia International Medical Technology Corporation	Zambia	Cholera vaccine	37 million	2024
Jiangsu Aidea Pharma, & Nanjing Pharmablock (in partnership with Fidscon Healthcare PLC)	Nigeria	HIV AVRs	100 million	2024
Wuhan Humanwell Hi-tech Industry Co. ltd	Mali	Injections, oral solid/liquid preparations	50 million	2015
	Ethiopia	Oral solid/liquid formulations and small-volume injections	20 million	2018
	Morocco	Psychiatric medication	TBD	2025

Source: Compiled by author from various sources.

One critical advantage for Chinese pharmaceutical companies expanding into Africa is the establishment of the AfCFTA. The AfCFTA creates a single liberalized African market for goods and services, aiming to enhance intra-African trade while deepening economic integration across the continent.^145^ For foreign companies, including Chinese pharmaceutical firms, this offers greater market access, reduced trade barriers, and simplified cross-border operations. By harmonizing regulatory frameworks and unifying standards across member states, the AfCFTA reduces the complexities of operating in multiple African markets.^146^

The growing presence of Chinese pharmaceutical companies in Africa, as illustrated in the above table, contributes to achieving the AfCFTA’s objectives by strengthening local production and enhancing access to affordable medicines. However, for these investments to deliver long-term benefits, it is crucial that they are localized to serve the interests of both Chinese companies and host African countries. A framework for the localization of these investments is essential to ensure mutual benefits.

The localization of Chinese enterprises in Africa has already proven beneficial for both parties.^147^ Many Chinese firms have recognized that localizing their operations improves relations with host governments, enhances their understanding of regional market dynamics, and facilitates smoother regulatory compliance. This, in turn, leads to increased efficiency and productivity.^148^ For African companies and host countries, localization enables transfer of technology and skill, leading to sustainability of local manufacturing.^149^

## IV. LOCAL PRODUCTION IN AFRICA: THE NEED FOR A HARMONIZED AND SUSTAINABLE APPROACH

As described above, there are multiple initiatives involved in local manufacturing across the continent, including those aimed at attracting investment in the area. This article argues that the success these initiatives hinges on their harmonization and sustainability. A harmonization approach emphasizes the need to streamline initiatives, particularly through pooled procurement, to ensure efficient resource utilization. In addition, the approach argues for regulatory alignment on critical issues like tariffs and packaging, as well as robust regional cooperation to support local production. In contrast, the sustainability approach advocates for diversifying production, addressing intellectual property challenges, and securing purchase guarantees for local manufacturers. The following section will detail each element of both approaches.

### IV.A. Streamlined Mandates and Pooled Procurement

In Africa, where financial and human resources are scarce and infrastructure is relatively underdeveloped, it is crucial for local manufacturing initiatives to collaborate rather than compete. Multiple continental, sub-continental, and global initiatives such as the APTF, PMPA, and PAVM all aim to enhance local medical products manufacturing and facilitate the transfer of technology.^150^ However, these initiatives often lack clear coordination and alignment in their mandates, leading to duplication of efforts and inefficient resource allocation. This can exacerbate human resource challenges and undermine long-term productivity.

To address these issues, initiatives should focus on pooling resources and aligning their objectives. For example, the PMPA aims to catalyze local pharmaceutical production to improve public health outcomes and economic benefits.^151^ The APTF seeks to foster collaboration between public and private sectors to build a resilient pharmaceutical industry.^152^ By harmonizing regulatory frameworks and fostering public–private partnerships, Africa can strengthen its pharmaceutical manufacturing capabilities and improve access to essential medicines. The recent report on the 24 Priority Medical Products and Roadmap for Regional Manufacturing in Africa provides a pivotal opportunity to leverage the roadmap to foster close coordination and alignment among these diverse local manufacturing initiatives.^153^ Effective coordination among these initiatives is essential to maximize their impact and ensure sustainable Africa’s pharmaceutical ecosystem.

The African Union General Assembly has established the APPM as a key component of the PAVM initiative, aimed at streamlining the procurement of medical products across the continent.^154^ Streamlined processes will reduce bureaucratic delays and inefficiencies, allowing for quicker response times in vaccine distribution, especially during health emergencies. A key step toward harmonizing existing initiatives is the recent decision to integrate the AfCFTA-Anchored Pharma Initiative’s pooled procurement mechanism (CCPM) into the APPM.^155^ Accordingly, the AfCFTA-Anchored Pharma Initiative will be transitioned into the start-up phase of the APPM, streamlining the CPPM and APPM under a unified continental framework. ^156^ By unifying these mechanisms, the initiative aims to enhance efficiency, reduce duplication of efforts, and strengthen Africa’s healthcare infrastructure.

### IV.B. Regional Regulatory Alignment and Capacity

As per WHO, only 7 percent of National Drug Regulatory Agencies (NDRAs) possess the necessary regulatory capacity to carry out essential tasks such as effective regulatory oversight and the capacity to provide approvals for local manufacturers while 90 percent of African countries possess minimal or no regulatory capacity.^157^ Out of 45 WHO African members that have their NDRAs benchmarked using the WHO Global Benchmarking Tools, only four countries (Ghana, Nigeria, South Africa, and Tanzania) have achieved the WHO Maturity 3 level, meeting the criteria for stable, well-functioning, and integrated regulatory system, while the remaining 41 members are either at Maturity level 1 or 2.^158^ This reflects a critical weakness in Africa’s national regulatory bodies in ensuring that medical products, either locally produced or imported, are safe, effective, and meet the required standard.

Enhancing regulatory capacity can be effectively achieved through the implementation of regionally aligned and standardized regulatory tools. Collaboration and standardization not only address complex regulatory challenges but also simplify the regulatory process. By harmonizing rules and regulations across regions, the burdens of intricate national requirements can be alleviated, which, in turn, enables national bodies to more effectively fulfill their responsibilities.

In this context, the AMRH, established in 2009 to address the lack of harmonization among NMRAs, has been actively working to standardize regulatory policies and guidelines.^159^ This harmonization not only streamlines processes but also encourages investment and innovation by improving efficiency and reducing barriers. AMRH also currently works with five RECs (EAC, ECCAS, ECOWAS, IGAD, and SADC), with the aim of implementing a common information management system for the registration of medicines, and building the regulatory capacity of NMRAs in member states of each REC.^160^ The close partnership between the AMRH and RECs is vital to enhance both regulatory capacity and GMPs. However, it is essential that this harmonization extends beyond medicine registration to include the reduction of barriers such as tariffs, fragmented tax and customs policies, logistical requirements, and enforcement against counterfeit medical products.

Another key area for regulatory collaboration, for instance, is in the standardization and harmonization of packaging requirements for medical products. For instance, the EU has adopted GMP packaging guideline for manufacturing and quality control of pharmaceutical products in the region.^161^ The EU Guide provides for key packaging and labeling requirements such as the materials used, the design of the package, clarity and legibility of the label, and environmental requirements.^162^ By establishing uniform packaging standards, countries can reduce the complexity of regulatory compliance, as manufacturers only need to meet a single set of criteria rather than navigating multiple national standards. This simplification can expedite the approval process and reduce the risk of non-compliance issues, ultimately ensuring safety and efficacy of pharmaceutical products across the region.^163^ Harmonized packaging also ensures that products are consistently protected against environmental factors, maintaining sterility and integrity throughout the supply chain.^164^ A similar framework for medical products in Africa will ensure clarity and enhance GMPs that ultimately contributes to attracting more producers to the sector.

Additionally, AfCFTA has the potential to significantly reduce regulatory hurdles that have long hindered pharmaceutical production across the continent. It is vital to leverage AfCFTA to eliminate unnecessary technical barriers to trade, including tariffs and restrictive tax policies. As member countries commit to progressively removing both tariff and non-tariff barriers, and cooperate on customs and investment matters,^165^ local manufacturing initiatives must capitalize on these developments.

### IV.C. Regional Cooperation for Infrastructure Development

A robust and reliable infrastructure is a sine quo non for local manufacturing to thrive, as it provides the foundational support necessary for efficient production and innovation. Governments must proactively support in enhancing pre-existing infrastructure, such as energy sources, transportation and communication networks, and the healthcare ecosystem, to create a conducive environment for manufacturing growth.^166^

Regional collaboration is also crucial for developing and maintaining infrastructure. One effective approach to achieving this is by enhancing regional collaboration on investments in cross-border supply chains. This strategy not only fosters deeper economic integration but also supports sustainable development by leveraging shared resources, expertise, and infrastructure. By improving cross-border supply chains, logistical barriers can be reduced, trade efficiency across borders can be increased, and supply chain resilience can be strengthened, which is especially crucial for the pharmaceutical and vaccine sector.^167^

Regional collaboration also facilitates the exchange of expertise, technology, and best practices, which can accelerate innovation and improve the quality of medical products.^168^ This is especially important for Africa, where the pharmaceutical and vaccine industry is highly concentrated in specific countries, such as South Africa, Nigeria, and Egypt, while many other African nations lack even a single local manufacturer.^169^ This uneven concentration of local producers exacerbates disparities in access to and affordability of essential medicines, leaving a significant portion of the population underserved. A well-developed and integrated regional infrastructure can help mitigate these challenges by facilitating the free movement of goods, services, skills, and technology. ^170^

For example, initiatives under the AfCFTA aim to harmonize regulatory frameworks, improve transportation networks, and promote shared manufacturing facilities, enabling better distribution of pharmaceutical and vaccine products to underserved areas.^171^ It is therefore critical to leverage the AfCFTA to consolidate regional markets for creating economies of scale. By integrating regional markets, Africa can overcome the challenges of small and fragmented markets, which have historically hindered local pharmaceutical and vaccine production. The AfCFTA offers a framework for harmonizing regulatory systems, reducing tariff and non-tariff barriers, and enhancing collaboration among countries. ^172^ This integration can lead to increased economies of scale for pharmaceutical and vaccine manufacturers, allowing them to produce more competitively and address the continent’s significant reliance on imported medical products.

In this context, the draft AfCFTA Protocol on Investment must be leveraged to support the development of Africa’s pharmaceutical and vaccine industry and broader economic integration. Adopted by the African Union Assembly in February 2023 and awaiting state signatures, the Protocol focuses on promoting and facilitating investment among African countries and companies.^173^ To achieve these objectives, investments must prioritize the enhancement of regional pharmaceutical and vaccine value chains and facilitate cross-border investments in local production, guided by comparative advantage. By aligning investments with the specific strengths of individual countries, such as raw material availability, manufacturing capacity, or technological expertise, the Protocol can help create a more efficient and equitable pharmaceutical and vaccine production network across the continent.

### IV.D. Addressing the Intellectual Property Conundrum: Lessons from the MPP

IPRs remain at the core of vaccine and pharmaceutical manufacturing as well as broader public health issues. IP holders, having invested significant resources into research, development, and production, maintain exclusive monopoly rights over their innovations. While these rights incentivize innovation, they also pose challenges for equitable access to life-saving vaccines and medical products, particularly in LMICs.^174^ For local production of pharmaceutical and vaccine products to occur, two main pathways are typically required: either through voluntary licensing agreements or by utilizing public health–related flexibilities and exceptions under international IP regimes.

Voluntary licensing involves agreements between IP holders and manufacturers, allowing the production of patented medicines or vaccines under specified terms, often in exchange for royalties. This approach has been used by the MPP as part of the WHO Vaccine Technology Transfer Hub. According to the MPP’s template for the technology transfer agreement, partners in LMICs are granted ‘a non-exclusive, royalty-free, non-sublicensable, non-transferable, irrevocable, fully paid-up, royalty-free licence under the Technology, the Afrigen Rights and the Biovac Rights to make, or have made, use, offer for sale, sell, have sold, export or import Product(s) in the Territory.’^175^ This is aimed at ensuring that LMICs can utilize the licensed technology without bearing the financial burden of royalties and, as a result, engage in affordable production and distribution of health products.

In addition, the partners also get access to any rights held by Afrigen and Biovac ‘… to make, or to have made, use, offer for sale, sell, have sold, export or import products in their respective territories and other LMICs.’^176^ This facilitates regional cooperation and enhances pharmaceutical value chains across LMICs since it ensures that the benefits of the technology transfer extend beyond a single country. In return, the partners must grant the MPP ‘… a non-exclusive, non-transferable, but sublicensable, irrevocable, fully paid-up, royalty-free, worldwide licence to practice and have practiced the data and the Inventions for the purposes of fulfilling its mission to facilitate the development and equitable access of health technologies in the Territory.’^177^

The issue of royalties for IP have not been without disagreements between the MPP and its partners in LMICs. For instance, Bio-Manguinhos, a selected technology transfer partner from Brazil, argues that as a non-profit, state-owned company, it is unjust for a technology developed with funding from the Brazilian government to be shared with other participating partners in LMICs, including for-profit entities, without any financial return or compensation.^178^ Because of the contention that public resources should not subsidize for-profit companies in other LMICs, the company is yet to sign the transfer of technology agreement with the MPP.^179^

Furthermore, MPP is granted ‘worldwide IP’ rights for innovations developed by program hosts in South Africa and partners in LMICs. In exchange, these partners receive limited IP rights that restricts them to manufacture, sell, and distribute products solely within their own territories and other LMICs.^180^ This imbalance in the scope of IP rights between the MPP and its partners has caused dissatisfaction among some partners. For example, Indonesia’s BioFarma, in its agreement with the MPP, successfully negotiated an exception, securing the right to market its products in high-income countries.^181^

Hence, it is crucial not only to encourage the voluntary participation of IP holders in technology transfer programs but also to establish mechanisms that ensure these initiatives are neither perceived as excessively burdensome for some participants nor as opportunities for free-riding by others for other partners in LMICs. Given the differing powers and interests of stakeholders in such programs, implementing clear guidelines and equitable frameworks is critical to fostering a collaborative environment that promotes innovation and shared benefits.^182^

In addition, countries must ensure that their IP laws fully leverage the flexibilities provided under the TRIPS Agreement. Effectively utilizing these flexibilities can help address IP-related challenges in public health, particularly by facilitating local vaccine manufacturing and enhancing technology transfer initiatives. For example, compulsory licensing allows governments to authorize the production of patented products without the patent holder’s consent, typically invoked during health emergencies or for public non-commercial use.^183^ The Doha Declaration affirms that WTO members have the right to grant compulsory licenses and the discretion to determine the conditions under which these licenses are issued.^184^ Furthermore, the TRIPS Agreement, particularly Articles 30 and 31, allows member countries to use patented products in response to public health issues. This is reinforced by the amendment to the TRIPS Agreement, known as Article 31bis, which permits WTO members with limited or no manufacturing capacity to import medicines under compulsory licensing, thereby removing the requirement to supply the domestic market as stipulated in Article 31.^185^ While challenges persist, such as ensuring effective technology transfer and navigating opposition from wealthier nations and pharmaceutical companies, these provisions can be strategically leveraged to enhance local production capabilities.^186^ This is especially crucial when voluntary licensing options are not feasible. Leveraging these flexibilities can help address IP-related challenges in public health, including enabling local manufacturing of vaccines and maximizing technology transfer initiatives.^187^

The AfCFTA must also effectively balance leveraging IP to address public health needs, such as local manufacturing, with protecting the interests of IP holders. In this regard, the draft AfCFTA IP Protocol, adopted in 2023 and currently awaiting ratification, identifies the promotion of local manufacturing of medical products as a core component of its IP policy. The Protocol specifically requires AfCFTA member states to ensure coherence across their national policies on IP, health, trade, industry, and innovation to advance local production of medical products.^188^ Additionally, the draft Protocol mandates that AfCFTA members fully exploit the flexibilities provided under the TRIPS Agreement, including public health–related exceptions.^189^ While the Protocol serves as a framework agreement that is expected to be supplemented by multiple annexes, it is essential to actively engage IP holders and the private sector. Their involvement is critical to effectively harnessing their expertise, resources, and participation in driving local manufacturing and facilitating technology transfer.

### IV.E. Diversification of Products and Technology Transfer Agreements

One of the post-pandemic challenges of local manufacturing has been that most companies exclusively focused on producing COVID-19 vaccines with no long-term-oriented and sustainable vision. As the pandemic subsided and demand for COVID-19 vaccines plummeted, the viability of these projects became increasingly uncertain. Moderna, a leading vaccine manufacturer, exemplifies this issue. In March 2022, it announced plans to build an mRNA COVID-19 vaccine facility in Kenya,^190^ finalizing an agreement with the Kenyan government in March 2023.^191^ However, by April 2024, Moderna announced that it has canceled its plan to set up mRNA COVID-19 vaccines manufacturing facility in Kenya.^192^ The company cites lack of demand for COVID-19 vaccines in Africa resulting in the unviability of the production facility. Specifically, the company notes the lack of orders from African governments ultimately leading to the cancelation of the project.^193^

This decision reflects on the post-pandemic challenges of establishing sustainable vaccine manufacturing and the need for a more sustainable approach to vaccine production that aligns with long-term healthcare needs and demand. It is crucial for local manufacturing to address the long-term needs of the region rather than solely focusing on immediate crises. Establishing a manufacturing facility and waiting for the next pandemic is not a viable strategy. Instead, local manufacturing should be designed to meet ongoing healthcare demands. This approach not only ensures sustainability but also supports continuous innovation and adaptation to emerging health challenges.

Furthermore, it is crucial to diversify technology transfer channels. Africa remains heavily reliant on technology transfers from non-African vaccine manufacturers to access active pharmaceutical ingredients and produce medical products. ^194^ Most technology transfer agreements between African manufacturers and non-African producers are limited to a single manufacturer. This dependency leaves the industry highly vulnerable to disruptions and supply chain issues. ^195^ Moreover, these agreements are predominantly focused on the manufacturing of COVID-19 vaccines.^196^ Given the declining demand for COVID-19 vaccines across the continent, a more diversified and long-term approach is essential to ensure resilience and sustainability.

Technology transfer is a complex and gradual process that requires careful execution. Its effectiveness depends on several factors, including proper planning and strategic partnerships, significant investment in materials and personnel, addressing intellectual property and licensing challenges, and ensuring effective policy and logistical coordination. Only by addressing these elements can Africa build a robust and self-reliant pharmaceutical manufacturing ecosystem.

### IV.F. Ensuring Demand through Conditional Purchase Guarantees

Like other manufacturing industries, vaccine and pharmaceutical manufacturing is shaped by the market forces of demand and supply. A steady supply of medical products relies heavily on a consistent and predictable demand, particularly from governments that play a key role in safeguarding and advancing public health. When producers face uncertainty regarding the purchase of their products, they are less likely to take on the financial risks associated with developing and manufacturing vaccines and pharmaceuticals. This hesitation is compounded by the substantial investments required for R&D, clinical trials, and the establishment of production facilities. Without clear and reliable demand commitments, the sustainability of pharmaceutical manufacturing becomes precarious.

Uncertain demand commitments from governments not only disincentivize investment in the pharmaceutical sector but also complicate manufacturers’ ability to secure technology transfer agreements. This underscores the importance of consistent government support, as seen in major vaccine-producing countries like China and India. These countries have historically backed local production through strong demand commitments, providing a stable foundation for their vaccine manufacturing industries.^197^

Local manufacturing in Africa also requires strong demand commitments from African governments, especially those that prioritize the purchase of local medical products in lieu of importing from abroad. At the same time, the PAVM framework proposes the establishment of an African Vaccines Procurement Pooling Mechanism (AVPPM) to ensure greater certainty of demand and volume stability for African manufacturers.^198^ However, the practical implementation of this initiative will be challenging. A recent decision by South Africa, host of the WHO technology transfer hub, to procure pneumococcal vaccines from India instead of its own company, Biovac, highlights these challenges.^199^ The decision was driven by cost considerations, as Indian vaccines were cheaper. This example underscores the critical need to align procurement policies in Africa with local production initiatives. Unless African governments and donor agencies prioritize sourcing vaccines from local manufacturers, the goal of sustainable vaccine access through local production will remain a myth.

In addition, it is crucial to closely study the lessons learned from the MPP and other procurement mechanisms. The MPP has encountered significant challenges in its efforts to enhance access to essential medicines through voluntary licensing. A major issue lies in the selection of companies based solely on their manufacturing capacity.^200^ In one instance, when licensing COVID-19 therapeutics, the MPP selected ~30 companies, many of which ultimately failed to produce the licensed medicines, and those that did often ceased production shortly thereafter.^201^ This oversupply creates a competitive environment that squeezes profit margins, as companies are compelled to undercut each other to secure sales. Consequently, manufacturers are often hesitant to produce unless they can ensure a high return on investment.^202^ A 2020 study found that while MPP’s approach increases generic competition and reduces prices, it also risks destabilizing markets when too many manufacturers enter without guaranteed demand.^203^

A key policy consideration to address this challenge is the implementation of purchase guarantees, which are advance purchase agreements with pharmaceutical companies to accelerate the development, manufacturing, and distribution of vaccines and other medical products. This approach has been successfully employed in countries like the USA during the COVID-19 pandemic, where the government has used advance purchase agreements to accelerate the development, manufacturing, and distribution of vaccines and other medical products.^204^ By providing assurance that manufactured products will be purchased, these agreements effectively mitigate financial risks for manufacturers, thereby encouraging sustained production and ensuring a stable supply of affordable medicines.^205^ In particular, to balance investments in local production with equitable access to vaccines and medical products, integrating conditional purchase guarantees with binding technology transfer requirements and tiered pricing mechanisms offers a viable pathway. This approach addresses both supply-side barriers (eg manufacturing capacity gaps) and demand-side risks (eg affordability constraints). Unfortunately, this critical element was lacking in MPP’s approach, which could have otherwise encouraged sustained production and ensured a stable supply of affordable medicines.

## V. CONCLUDING REMARKS

The imperative for local vaccine and pharmaceutical production in Africa has never been clearer, particularly in the wake of the COVID-19 pandemic. The continent’s heavy reliance on imports has exposed critical vulnerabilities, underscoring the need for a harmonized and sustainable approach to healthcare manufacturing. By investing in local production capabilities, African nations can enhance their self-reliance, improve access to essential medical products, and mitigate the risks associated with global supply chain disruptions.

Continental initiatives such as the PMPA, PAVM, and other regional and global collaborations are vital in establishing a strong pharmaceutical ecosystem. However, for these initiatives to be effective, it is essential that they are harmonized and sustainable.

Regulatory harmonization is critical for enhancing the local manufacturing sector. Currently, many African countries face complex regulatory environments that hinder the establishment of competitive manufacturing capabilities. Streamlining regulatory processes and establishing common standards will facilitate easier market entry for local producers and encourage investment in the sector. Initiatives like the AMRH aim to address these challenges by promoting standardized regulatory policies across member states.

Also, infrastructure development is paramount. A robust healthcare infrastructure, including reliable energy sources, transportation networks, and skilled labor, is essential for the success of local manufacturing initiatives. Regional cooperation on infrastructure investments can enhance the overall efficiency and effectiveness of pharmaceutical production across the continent.

Moreover, addressing intellectual property challenges is essential for fostering innovation and technology transfer. The MPP has shown that strategic licensing agreements can enhance access to life-saving technologies. African nations must leverage these frameworks to facilitate local production and ensure that the benefits of technological advancements are equitably distributed.

Finally, there is a pressing need to secure consistent demand for locally produced vaccines and pharmaceuticals. Government commitments to prioritize local procurement can create a stable market environment, encouraging manufacturers to invest in production capabilities. The establishment of an AVPPM can further enhance demand certainty and ensures that local producers have reliable outlets for their products. In addition, accessing regional and global markets is important for sustainability. For instance, African manufacturers that want to access the GAVI market need to fulfill WHO prequalification. Such qualification would mean more developing time and more investments to meet global quality standards.

## Footnotes

1 Tolulope Anthony Adekola & Bryan Mercurio, *mRNA Technology Transfer Hub and Intellectual Property: Towards a More Equitable ad Sustainable Model*, 2025 World Trade Review 1–19, 2.

2 Landry Signe, *The Potential of Manufacturing and Industrialization in Africa: Trends, Opportunities, and Strategies*, Africa Growth Initiative at Brookings, 2018, 7.

3 G7ITALIA, *Report: Enhancing Pharmaceutical Manufacturing Capacity in Africa: Current Scenario and Future Perspectives for G7 Members*, October 14, 2024, https://www.g7italy.it/wp-content/uploads/Report_Pharmaceutical-Manufacturing-in-Africa_20241014.pdf (accessed Jan. 27, 2025).

4 Pamela Steele et al., *A Case for Local Pharmaceutical Manufacturing in Africa in Light of the COVID-19 Pandemic*, July 2020, https://www.childhealthtaskforce.org/sites/default/files/2021-08/20200715_LocalPharmaManufacturingInAfrica.pdf (accessed Feb. 1, 2025).

5
*Ibid*.

6 Airfinity, *COVID-19 Intel Report*, December 16, 2021, https://www.ifpma.org/wp-content/uploads/2023/01/i2023_Airfinity_COVID-19_Intel_Report_16December2021.pdf (accessed Feb. 1, 2025).

7 Roxana Philip et al., *Global Challenges to Public Healthcare Systems during the COVID-19 Pandemic: A Review of Pandemic Measures and Problems*, 2022 12 Journal of Personalized Medicine 1295.

8 Forbes, ‘The Ukraine-Russia War’s Impact on the Supply Chain: Why MRO Optimization is a Top Priority’, May 12, 2022, https://www.forbes.com/sites/paulnoble/2022/05/12/the-ukraine-russia-wars-impact-on-the-supply-chain-why-mro-optimization-is-a-top-priority/ (accessed Feb. 2, 2025).

9 Bryan Mercurio, *The Demise of Globalization and Rise of Industrial Policy: Caveat Emptor*, 2023 23 World Trade Review 242–250.

10
*Ibid*. See also Valentine Millot & Lukasz Rawdanowicz, *The Return of Industrial Policies: Policy Considerations in the Current Context*, OECD Economic Policy Papers No. 34, 2024, https://www.oecd.org/en/publications/the-return-of-industrial-policies_051ce36d-en.html (accessed Feb. 7, 2025).

11 See for instance, Philip Schellekens, *Local Vaccine Manufacturing Pays Off*, September 7, 2023, https://pandem-ic.org/local-vaccine-manufacturing-pays-off/ (accessed Feb. 2, 2025).

12 WHO, *Immunization*, https://www.afro.who.int/health-topics/immunization accessed on Jan. 29, 2025.

13 WHO, ‘Business Case for WHO Immunization Activities on the African Continent 2018–2030’, WHO Regional Office for Africa, 2018, page 5.

14 WHO Regional Committee for Africa, ‘Framework for Strengthening Local Production of Medicines, Vaccines and Other Health Technologies in the WHO African Region 2025–2035: Report of the Secretariat’, WHO Doc. AFR/RC74/6, Brazzaville, Democratic Republic of Congo, July 22, 2024.

15 GAVI: The Vaccine Alliance, *Expanding Sustainable Vaccine Manufacturing in Africa: Priorities for Support*, November 2022, https://www.gavi.org/sites/default/files/document/2022/Gavi-Expanding-Sustainable-Vaccine-Manufacturing-in-Africa-2022.pdf (accessed Feb. 2, 2025). See also World Economic Forum, *Ramping Up Africa’s Vaccine Manufacturing Capability is Good for Everyone. Here’s Why*, October 17, 2022, https://www.weforum.org/stories/2022/10/ramping-up-africa-s-vaccine-manufacturing-capability-is-good-for-everyone-heres-why/ (accessed Feb. 2, 2025).

16 Emmanuel Lamptey et al., *COVID-19 Vaccines Development in Africa: A Review of Current Situation and Existing Challenges of Vaccine Production*, 2022 11 Clinical and Experimental Vaccine Research 82, 85. See also Toyin Abiodun et al., *Vaccine Manufacturing in Africa: What It Takes and Why It Matters*, Tony Blair Institute for Global Change, 2021, https://institute.global/insights/public-services/vaccine-manufacturing-africa-what-it-takes-and-why-it-matters (accessed Feb. 2, 2025).

17
*Ibid*. See also Zainab Usman & Juliette Ovadia, *Is There Any Covid Vaccine Production in Africa?*, September 13, 2021, https://carnegieendowment.org/posts/2021/09/is-there-any-covid-19-vaccine-production-in-africa?lang=en (accessed Feb. 4, 2025).

18 See also Obi Peter Adigwe et al., *Current State and Challenges of Local Production of Vaccines in Nigeria*, 2024 3 Public Health Challenges 1.

19 World Economic Forum, *AfCFTA: A New Era for Global Business and Investment in Africa: Insight Report*, January 2023, 14.

20 G7ITALIA Finance Task, note 3 above.

21
*Ibid*. The eight countries are Algeria, Egypt, Ghana, Kenya, Morocco, Nigeria, South Africa, and Tunisia.

22
*Ibid*. See also *Making Africa’s Pharmaceutical Ambitions a Reality*, A report by Development Reimagined, August 2022, https://developmentreimagined.com/wp-content/uploads/2022/08/afr_pharm_report_final-_format_oo_29jul.pdf (accessed Feb. 3, 2025).

23 Africa Development Bank Group, *A New Frontier for African Pharmaceutical Manufacturing Industry*, 2022, https://www.afdb.org/sites/default/files/documents/publications/pharmaceutical_industry_and_vaccine_production_web.pdf (accessed Feb. 4, 2025).

24 Cara Lynn Kim et al., *Overcoming Vaccine Inequities and Research Gaps in Africa: Challenges and Opportunities Identified During the COVID-19 Pandemic*, 2025 80(1) Clinical Infectious Diseases, S9.

25
*Ibid*. See also Pamela Steele et al. note 4 above.

26 African Union, ‘Pharmaceutical Manufacturing Plan for Africa: Business Plan’, July 2007.

27
*Ibid*, paragraph 5.

28
*Ibid*, paragraphs 17–20.

29 African Union, *Pharmaceutical Manufacturing Plan for Africa: Business Plan*, 2012, https://www.nepad.org/publication/pharmaceutical-manufacturing-plan-africa (accessed Jan. 29, 2025).

30
*Ibid*.

31 AUDA-NEPAD, *24 Priority Medical Products and Road-Map for Regional Pharmaceutical Manufacturing in Africa Report, January 2025*, https://www.nepad.org/publication/24-priority-medical-products-and-roadmap-regional-manufacturing-africa (accessed Feb. 8, 2025).

32
*Ibid*, page 15.

33 The list for each category includes the following: **High Need-High Market** (Antibiotics: Amoxicillin, Pain management: Paracetamol, HIV/AIDS: HIV tests, Cabotegravir, Tenofovir + Lamivudine + Dolutegravir (TLD), Antimalarials: mRDTs, Artemether + lumefantrine, Sulfadoxinepyrimethamine, TB: Bedaquiline), **Growing Need-High Market** (Stroke: Atorvastatin, Injuries: Intravenous fluids, Diabetes: Insulin analogues, Metformin, Ischemic Heart Disease/Hypertension: Amlodipine, Valsartan), **Low Need-High Market** (Cancer: Docetaxel), **High Need-Low Market** (Maternal health: Oxytocin, Misoprostol, Magnesium sulfate, Newborn: Birth asphyxia and birth trauma—Medical Oxygen, Child health: Antidiarrheals: Oral rehydration salts + Zinc sulfate, Rotavirus vaccine), **Growing Need-Low Market** (Sickle cell disease: Hydroxyurea), and **Low Need-Low Market** (NTDs: Praziquantel).

34
*Ibid*, page 89.

35
*Ibid*.

36 Janet Byaruhanga, *The Pharmaceutical Manufacturing Plan for Africa*, August 24, 2020, https://www.nepad.org/news/pharmaceutical-manufacturing-plan-africa (accessed Aug. 25, 2025).

37 Africa Centre for Disease Control and Prevention, *Partnerships for African Vaccine Manufacturing (PAVM) Framework for Action*, 2022, page 8.

38
*Ibid*, page 9.

39
*Ibid*.

40
*Ibid*.

41
*Ibid*, pages 74, 81 & 88.

42 Africa CDC, *The 2^nd^ Vaccines and Other Health Products Manufacturing Forum for African Union Member States: Communique*, February 2025, https://africacdc.org/news-item/communique-the-2nd-vaccines-and-other-health-products-manufacturing-forum-for-african-union-member-states/ (accessed Feb. 12, 2025).

43
*Ibid*.

44 Africa CDC, *Clinton Health Access Initiative & PATH, Africa Vaccine Manufacturing Mapping: Supply and Demand Landscape*, October 2024. See also Africa CDC, *Africa’s Progress Towards Sustainable Local Manufacturing Health Products*, February 11, 2025, https://africacdc.org/news-item/africas-progress-towards-sustainable-local-manufacturing-health-products/ (accessed Feb. 14, 2025).

45
*Current and Planned Vaccine Manufacturing in Africa: Results from a joint assessment by Africa CDC, CHAI, and PATH*, August 2023, https://media.path.org/documents/AVM_mapping_briefing_paper_091823_FINAL.pdf?_gl=1*1cxxj7h*_gcl_au*MzM1Mjc0NzczLjE3Mzk1MDk2MDE.*_ga*OTU0NTM5NjkxLjE3Mzk1MDk2MDE.*_ga_YBSE7ZKDQM*MTczOTUwOTYwMS4xLjEuMTczOTUwOTczMi40MS4wLjA (accessed Feb. 11, 2025).

46 African Development Bank Group, *Africa gets shot in the arm as African Pharmaceutical Technology Foundation gets underway*, December 18, 2023, https://www.afdb.org/en/news-and-events/press-releases/africa-gets-shot-arm-african-pharmaceutical-technology-foundation-gets-underway-67262 (accessed Feb. 04, 2024).

47 African Pharmaceutical Technology Foundation: Our Programs, https://www.aptfound.org/our-programs (accessed Feb. 04, 2025).

48 See Morris Kingsley Ode, *The Covid-19 Pandemic and Access to Medicines in Africa: A Critical Review of the African Pharmaceutical Technology Foundation*, AfronomicsLaw, March 21, 2024, https://www.afronomicslaw.org/category/analysis/covid-19-pandemic-and-access-medicines-africa-critical-review-african (accessed Feb. 12, 2025).

49 African Union, *Statement by H.E. Dr Monique Nsanzabaganwa, Deputy Chairperson of the African Union Commission: An International Conference on Innovation, Intellectual Property, and Technology Transfer in Africa’s Pharmaceutical Sector*, March 25, 2024, Addis Ababa, Ethiopia, https://au.int/sites/default/files/speeches/43767-sp-DCP_Statement_-_ATPF_Conference.pdf (accessed Feb. 14, 2025).

50
*Ibid*. See also AfCFTA Draft IPRs Protocol, Article 23.

51
Carlos Correa, Intellectual Property Rights, the WTO and Developing Countries: The TRIPS Agreement and Policy Options, Zed Books, 2020, 89.

52 Bryan Mercurio, Tolulope Anthony Adekola & Chimdessa Fekadu Tsega, *Pharmaceutical Patent Law and Policy in Africa: A Survey of Selected SADC Member States*, 2023 43 Legal Studies 331, 338.

53
*Ibid*.

54 Treaty for the Establishment of the African Medicines Agency (AMA Treaty), February 11, 2019.

55
https://amrh.nepad.org/, (accessed Feb. 20, 2025).

56 AMA Treaty, Article 8. The term ‘Medical products’ includes vaccines, medicines, blood and blood products, diagnostics, and medical devices. See the AMA Treaty, Article 2.

57
*Ibid*, Article 3.

58 Molly Unoh Ogbodum et al., *African Medicines Agency: How It Will Change the Landscape of Medicines in Africa*, 2023 2 Public Health Challenges 1.

59 AUDA-NEPAD, *African Medicines Agency: What’s AMA’s Role?*, https://www.nepad.org/microsite/african-medicines-agency-ama (accessed Feb. 15, 2024).

60 AMA Treaty, Article 6.

61 Economic Commission for Africa, *AfCFTA-Anchored Pharma Initiative*, https://www.uneca.org/afcfta-anchored-pharma-initiative#:~:text=The%20AfCFTA%2D%20Anchored%20Pharmaceutical%20Initiative,Poverty%20and%20Social%20Policy%20Divisions (accessed Feb. 16, 2025). See also Economic Commission for Africa, *Roadmap to operationalize Pharma Initiative into start-up Phase of the African Pooled Procurement Mechanism adopted*, May 16, 2024, https://www.uneca.org/stories/roadmap-to-operationalize-pharma-initiative-into-start-up-phase-of-the-african-pooled (accessed Feb. 16, 2025).

62
*Ibid*.

63 Economic Commission for Africa, *The Pharma Initiative Transformative Journey*, https://www.uneca.org/sites/default/files/afcfta-pharma-initiative/final_eng_pharma-initiative-transformative-journey-brochure_10022024_all.pdf (accessed Feb. 16, 2025).

64
*Ibid*.

65
*Ibid*.

66 Economic Commission for Africa, AfCFTA-Anchored Pharma Initiative, *Preliminary survey report on the assessment of manufacturing capacity status, good manufacturing practice and adherence to environmental, health and safety standards among selected local pharmaceutical manufacturers in 10 pilot countries*, May 2023, https://repository.uneca.org/bitstream/handle/10855/49949/b12037862.pdf?sequence=1&isAllowed=y (accessed Feb. 20, 2025).

67 Economic Commission for Africa, *CONCEPT NOTE: AfCFTA-anchored Pharmaceutical Initiative Ministerial Meeting on the Endorsement of the Centralized Pooled Procurement Mechanism and Signature to the Agreement*, 14–16 May 2024, Mombasa, Kenya, https://www.uneca.org/ecaevents/sites/default/files/resources/documents/afcfta-anchored-pharmaceutical-initiative/concept_note_afcfta-anchored_pharma_initiative_ministerial_conference.pdf (accessed Feb. 20, 2025).

68 East Africa Community, *East African Community Regional Pharmaceutical Manufacturing Plan of Action (2012–2016)*, March 2018.

69
*Ibid*, page 9.

70
*Ibid*.

71 East Africa Community, *2^nd^ EAC Regional Pharmaceutical Manufacturing Plan of Action 2017–2027 (EAC-RPMPOA)*, March 2018, 27.

72
*Ibid*. See also Olugbenga Ajani Olatunji, *Regional Approach to Boosting Local Pharmaceutical Manufacturing Capacity: A Critique of the East African Community Pharmaceutical Manufacturing Plan of Action*, 2024 73 GRUR International 1036.

73
*Ibid*, 35.

74 The following EAC countries are identified as Least Developed Countries (LDCs): the Democratic Republic of Congo, Kenya, Burundi, Rwanda, Somalia, South Sudan, Tanzania, and Uganda. See United Nations, Office of the High Representative for the Least Developed Countries, *Landlocked Developing Countries and Small Island Developing States: Least Developed Countries*, https://www.un.org/ohrlls/content/list-ldcs accessed on Aug. 26, 2025.

75 EAC-RPMPOA, 32.

76 East African Community, *EAC Regional Intellectual Property Policy on the utilisation of public health-related WTO-TRIPS flexibilities and the approximation of national intellectual property legislation*, 2013. See also the East African Community Health Protocol on Public Health Related WTO-TRIPS Flexibilities, 2013.

77 Olugbenga Ajani Olatunji, note 72 above.

78
*AUDA-NEPAD, EAC-MRH REC Project*, https://amrh.nepad.org/amrh-recs/eac-mrh-rec-project accessed on Aug. 25, 2025.

79 Olugbenga Ajani Olatunji, note 75 above.

80 EAC-RPMPOA, page 36.

81
*ECOWAS West African Health Organization, Regional Pharmaceutical Plan (ERPP)*, WAHO/Technical document/04.14, Dobo-Dioulasso, Burkina Faso, April 2014.

82 ECOWAS West African Health Organization, *A Regional GMP Roadmap Framework for the Pharmaceutical Manufacturing Industry in ECOWAS*, 2019, page 1.

83 ECOWAS ERPP, pages 13–16.

84
*Ibid*.

85
*Ibid*, pages 16–21.

86 ECOWAS ERPP Resolution, page 3.

87 ECOWAS West African Health Organization, *A Regional GMP Roadmap Framework for the Pharmaceutical Manufacturing Industry in ECOWAS*, 2019, https://hub.unido.org/sites/default/files/publications/ECOWAS%20GMP%20ROADMAP%20FRAMEWORK_English_ebook_0.pdf (accessed Feb. 17, 2025).

88 WHO, *The mRNA Vaccine Technology Transfer Hub*, https://www.who.int/initiatives/the-mrna-vaccine-technology-transfer-hub (accessed Feb. 16, 2025).

89
*Ibid*.

90
*Ibid*.

91 Medicines Patents Pool, https://medicinespatentpool.org/what-we-do/mrna-technology-transfer-programme (accessed Feb. 16, 2025). See also Afrigen, *The Centre for mRNA technology development and transfer*, https://www.afrigen.co.za/mrna-hub/ (accessed Feb. 16, 2025).

92 Medicines Patents Pool, *mRNA Vaccine Technology Transfer Agreement: Template*, Article 3(4), available at https://medicinespatentpool.org/what-we-do/mrna-technology-transfer-programme/agreements#pills-Partners--Agreements (accessed Feb. 16, 2025).

93 Medicines Patents Pool, *mRNA Technology Transfer Programme Brochure*, May 2024, https://medicinespatentpool.org/uploads/2024/07/mRNA_Brochure_17x22-cm_MAY-2024_EN.pdf accessed on February 16, 2025.

94 Medicines Patents Pool, https://medicinespatentpool.org/what-we-do/mrna-technology-transfer-programme/agreements#pills-Partners--Agreements accessed on February 16, 2024. The partners based in Africa are Biovac of South Africa, Biogeneric Pharma of Egypt, Biovaccines Nigeria, Institut Pasteur de Dakar of Senegal, and Institut Pasteur de Tunis of Tunisia. Biovax of Kenya is also among the African partners, but has not yet concluded the technology transfer agreement.

95 MPP Brochure, note 93 above.

96 MPP, note 91 above.

97
*Ibid*.

98 Afrigen, *The Centre for mRNA Technology Development and Transfer*, https://www.afrigen.co.za/mrna-hub/ (accessed Feb. 16, 2025).

99 ‘GAVI’, https://www.gavi.org/ (accessed Feb. 6, 2025).

100 ‘GAVI: Programmes and Impact’, https://www.gavi.org/programmes-impact/types-support/regional-manufacturing-strategy/avma (accessed Feb. 6, 2025).

101 GAVI, *African Vaccine Manufacturing Accelerator Guidance for Manufacturers*, June 20, 2024, https://www.gavi.org/sites/default/files/support/AVMA-Guidance-for-Manufacturers.pdf (accessed Feb. 6, 2025).

102
*Ibid*.

103
*Ibid*.

104
*Ibid*. see also WHO, *Prequalification of Medical Products*, https://extranet.who.int/prequal/vaccines/vaccines-eligible-who-prequalification (accessed Feb. 6, 2025).

105
*Ibid*.

106 Michael Conway et al., *Should sub-Saharan Africa make its own drugs?*, McKinsey & Company, January 10, 2019, https://www.mckinsey.com/industries/public-sector/our-insights/should-sub-saharan-africa-make-its-own-drugs (accessed Feb. 8, 2025).

107 Forum on China-Africa Cooperation (FOCAC), https://www.focac.org/eng/ltjj_3/zfhxxdwyh/ (accessed Feb. 9, 2025).

108 See for instance, Beyond The Horizon, *China-Africa Relations in Transition: The 2024 FOCAC Summit and Its Implications*, September 18, 2024, https://behorizon.org/china-africa-relations-in-transition-the-2024-focac-summit-and-its-implications/ accessed on Jan. 28, 2025.

109 Xinhua News, *Belt and Road Incorporated into CPC Constitution*, October 24, 2017, http://www.xinhuanet.com/english/2017-10/24/c_136702025.htm (accessed Feb. 4, 2025). See also *The Belt and Road Initiative: A Key Pillar of the Global Community of Shared Future*, The State Council Information Office of the People’s Republic of China, October 2023, https://www.beltandroadforum.org/english/n101/2023/1010/c124-895.html (accessed Feb. 4, 2025).

110 Shaoyu Yuan, *The Health Silk Road: A Double-Edged Sword? Assessing the Implications of China’s Health Diplomacy*, (2023) 4 World 333.

111 China Daily, *WHO, China sign pact establishing ‘health Silk Road*, January 19, 2017, https://www.chinadaily.com.cn/business/2017wef/2017-01/19/content_27993857.htm accessed on Jan. 23, 2025.

112 The State Council of the People’s Republic of China, *Visions and Actions on Jointly Building Silk Road Economic Belt and Road and 21^st^ Century Maritime Road*, Issued by the National Development and Reform Commission, Ministry of Foreign Affairs, and the Ministry of Commerce of the People’s Republic of China, with State Council Authorization, March 2015, https://2017.beltandroadforum.org/english/n100/2017/0410/c22-45.html accessed on Jan. 21, 2025.

113
*Ibid*.

114 National Health Commission of People’s Republic of China, *Beijing Communique of the Belt and Road Health Cooperation & Health Silk Road*, August 18, 2017, see Beijing Communiqué of The Belt and Road Health Cooperation & Health Silk Road accessed on Jan. 23, 2025.

115
*Ibid*.

116 Ministry of Commerce of the People’s Republic of China, *China-Africa Cooperation Vision 2035*, December 10, 2021, https://www.focac.org/eng/zywx_1/zywj/202201/t20220124_10632442.htm accessed on Jan. 23, 2025.

117
*Ibid*.

118 Ministry of Foreign Affairs of the People’s Republic of China, *Forum on China-Africa Cooperation Beijing Action Plan (2025–2027)*, September 05, 2024, https://www.mfa.gov.cn/eng/xw/zyxw/202409/t20240905_11485719.html accessed on Jan. 24, 2025.

119
*Ibid*, paragraph 10.1.4 & 10.1.5.

120 The State Council Information Office of the People’s Republic of China, *Full Text: Joint Statement of the High Level Video Conference on Belt and Road International Cooperation: Combating COVID-19 with Solidarity*, June 19, 2000, http://english.scio.gov.cn/beltandroad/2020-06/19/content_76349411.htm accessed on Jan. 23, 2025.

121 Ministry of Foreign Affairs of the People’s Republic of China, *Joint Statement of the Extraordinary China-Africa Summit on Solidarity Against COVID-19*, June 17, 2020, https://www.mfa.gov.cn/eng/zy/gb/202405/t20240531_11367415.html accessed on Jan. 23, 2025.

122 Yizhou He, *China-Africa Collaboration in the Health Sector under the Epidemic of COVID-19*, in Mammo Muchie et al. (eds.), China-Africa Science, Technology and Innovation Collaboration, Springer, 2024, 473–487.

123 The State Council Information Office of the People’s Republic of China, *China has provided over 2.2 billion COVID Vaccine Doses to the World*, May 12, 2022, http://english.scio.gov.cn/internationalexchanges/2022-05/12/content_78214119.htm (accessed Feb. 04, 2025).

124 See for instance BBC News, *Covid-19 Vaccines: Has China made more than other countries combined?*, October 10, 2021, https://www.bbc.com/news/58808889 (accessed Feb. 4, 2025).

125 Ministry of Foreign Affairs of the People’s Republic of China, *Xi Jinping Chairs the Extraordinary China-Africa Summit On Solidarity Against COVID-19 and Gives a Keynote Speech*, June 18, 2022, https://www.mfa.gov.cn/eng/wjb/zzjg_663340/fzs_663828/xwlb_663830/202406/t20240606_11392891.html (accessed Feb. 25, 2025).

126
*Ibid*.

127 Bridge Consulting, *China COVID-19 Vaccine Tracker*, last updated on December 28, 2022, https://bridgebeijing.com/our-publications/our-publications-1/china-covid-19-vaccines-tracker/ accessed on Jan. 25, 2025.

128
*Ibid*. Saidal of Algeria and Vacsera of Egypt manufactured Sinovac vaccine while Sotherma of Morocco manufactured Sinopharm vaccine.

129
*Ibid*.

130 World Health Organization, *COVAX: Working for global equitable access to COVID-19 vaccines*, https://www.who.int/initiatives/act-accelerator/covax#:~:text=COVAX%20was%20the%20vaccines%20pillar,tests%2C%20treatments%2C%20and%20vaccines accessed on Jan. 30, 2025.

131 Washington Post, *China Joins WHO-backed vaccine plan that the White House spurned*, October 9, 2020, https://www.washingtonpost.com/world/asia_pacific/coronavirus-vaccine-china-covax-who/2020/10/08/cf4e1e96-09d2-11eb-8719-0df159d14794_story.html accessed on Jan. 29, 2025. See also Liyong Jin & Liangtao Liu, *China’s Engagement in COVAX: From Hesitation to Active Participation*, 2021 3 Chinese Journal of International Review (online).

132 GAVI: The Vaccine Alliance, *China pledges US$100 million toward equitable access to COVID-19 vaccines for lower-income countries*, August 6, 2021, https://www.gavi.org/news/media-room/china-pledges-us-100-million-towards-equitable-access-covid-19-vaccines (accessed Feb. 4, 2025).

133 Veronika Blablove, *Assessing China’s Vaccine Diplomacy*, September 29, 2022, https://chinaobservers.eu/assessing-chinas-vaccine-diplomacy/ (accessed Feb. 4, 2025). See also AIDDATA, *Mapping China’s COVID-19 aid footprint: What does China’s billion-dollar campaign tell us about future pandemic diplomacy?*, December 19, 2024, https://www.aiddata.org/blog/mapping-chinas-covid-19-aid-footprint (accessed Feb. 4, 2025).

134 China Africa Research Initiative, *Chinese Investment in Africa 2003–2022*, https://www.sais-cari.org/chinese-investment-in-africa (accessed Feb. 20, 2025). See also Xuewu Gu et al., ‘China’s Engagement in Africa: Activities, Effects and Trends’, CGS Global Focus, June 2022, 2.

135 African Union, *Agenda 2063: The Africa We Want*, September 2015, https://au.int/sites/default/files/documents/33126-doc-framework_document_book.pdf (accessed Feb. 20, 2025).

136 Ministry of Foreign Affairs of the People’s Republic of China, *Forum on China-Africa Cooperation Beijing Action Plan (2025–2027)*, September 5, 2024, https://www.mfa.gov.cn/eng/xw/zyxw/202409/t20240905_11485719.html (accessed Feb. 19, 2025).

137
*Ibid*, paragraph 10.1.1.

138
*Ibid*, paragraph 10.1.5.

139 The State Council of the People’s Republic of China, *FULL TEXT: Keynote address by Chinese President Xi Jinping at opening ceremony of 2024 FOCAC summit*, September 5, 2024, https://english.www.gov.cn/news/202409/05/content_WS66d964bdc6d0868f4e8eaa07.html (accessed Feb. 19, 2025).

140 See for instance, South China Morning Post, *Chinese pharmaceutical firms expand to Africa under ‘health silk road*, October 14, 2024, https://www.scmp.com/news/china/diplomacy/article/3282304/chinese-pharmaceutical-firms-expand-africa-under-health-silk-road (accessed Feb. 19, 2025). Nikkei Asia, *Chinese drugmakers march into Africa*, June 17, 2023, https://asia.nikkei.com/Spotlight/Caixin/Chinese-drugmakers-march-into-Africa (accessed Feb. 19, 2025).

141 Current and Planned Vaccine Manufacturing in Africa, note 45 above.

142 Afrexim Bank, *Industry Outlook: Pharmaceuticals and Healthcare*, 2022, 4.

143 African Development Bank, *;A New Frontier for African Pharmaceutical Manufacturing Industry*, 2022.

144 China Global South Project, *Can China Help Build Africa’s Nascent Pharmaceutical Sector?*, October 16, 2020, https://chinaglobalsouth.com/analysis/can-china-help-build-africas-nascent-pharmaceutical-sector/ (accessed Feb. 20, 2025). See also United National Conference on Trade and Development (UNCTAD), *The Potential of Africa to Capture Technology-Intensive Global Supply Chains: Economic Development in Africa Report 2023*, 2023, 9.

145 AfCFTA Agreement, Articles 3&4. See also, UNCTAD, *Reaping the Potential Benefits of the African Continental Free Trade Area for Inclusive Growth*, Economic Development in Africa Report, 2021, 45.

146
*Ibid*. See also World Economic Forum, *In Africa’s free trade area, investment in pharmaceuticals means impact and profit*, March 28, 2023, https://www.weforum.org/stories/2023/03/in-africa-s-free-trade-area-investment-in-pharmaceuticals-means-impact-and-profit/ (accessed Feb. 20, 2025).

147 Barry Sautman & Yan Hairong, *Localizing Chinese Enterprises in Africa: from Myths to Policies*, HKUST IEMS Thought Leadership Brief No. 5, February 2015, https://iems.ust.hk/tlb5 (accessed Feb. 14, 2025).

148
*Ibid*. See also Belt and Road Portal, *China Focus: Chinese pharmaceutical companies make inroads into Africa amid growing health cooperation*, October 25, 2024, https://eng.yidaiyilu.gov.cn/p/07J7DO43.html (accessed Feb. 27, 2025).

149 United Nations Economic Commission for Africa, *Leveraging the AfCFTA to complement localized production and pooled procurement of healthcare products for reducing healthcare costs and poverty eradication*  https://social.desa.un.org/sites/default/files/inline-files/KARONGA_Paper.pdf (accessed Feb. 27, 2025).

150 See also Morris Kingsley Odeh, *The COVID-19 Pandemic and Access to Medicines in Africa: A Critical Review of the African Pharmaceutical Technology Foundation (APTF)*, AfronomicsLaw, March 21, 2024, https://www.afronomicslaw.org/category/analysis/covid-19-pandemic-and-access-medicines-africa-critical-review-african (accessed Feb. 20, 2025).

151 PMPA Business Plan, note 29 above.

152 APTF, note 47 above.

153 AUDA-NEPAD, *24 Priority Medical Products and Roadmap for Regional Manufacturing in Africa*, January 2025, https://www.nepad.org/publication/24-priority-medical-products-and-roadmap-regional-manufacturing-africa (accessed Feb. 20, 2025). For the list of the 24 priority medical products, see note 32 above.

154 Africa CDC, *The 2^nd^ Vaccines and Other Health Products Manufacturing Forum for African Union Member States: Communique*, February 2025, https://africacdc.org/news-item/communique-the-2nd-vaccines-and-other-health-products-manufacturing-forum-for-african-union-member-states/ (accessed Feb. 12, 2025).

155 Economic Commission for Africa, *Roadmap to operationalize Pharma Initiative into start-up Phase of the African Pooled Procurement Mechanism adopted*, May 16, 2024, https://www.uneca.org/stories/roadmap-to-operationalize-pharma-initiative-into-start-up-phase-of-the-african-pooled (accessed Feb. 20, 2025).

156 Economic Commission for Africa, ‘Concept Note’ note 67 above.

157 WHO, ‘WHO Global Benchmarking Tool (GBT) for Evaluation of National Regulatory System of Medical Products: Revision VI’, 2021, https://iris.who.int/bitstream/handle/10665/341243/9789240020245-eng.pdf?sequence=1 (accessed Feb. 10, 2025).

158 WHO African Region, *Framework for Local Production*, note 14 above.

159 AUDA-NEPAD, https://amrh.nepad.org/amrh-microsite/who-we-are. See also World Economic Forum, note 19 above, 14.

160
*Ibid*.

161 European Commission, *Guideline on the Packaging Information of Medicinal Products for Human Use Authorized by The Union: Final Revision 14.8*, September 2023.

162
*Ibid*.

163 See for instance, *Hong Kong Guide to Good Manufacturing Practice for the Secondary Packaging of Pharmaceutical Products*, December 2014.

164 See also, Flexan, *The Importance of Medical Device Packaging*, https://www.flexan.com/en-hk/news/the-importance-of-medical-device-packaging (accessed Feb. 28, 2025).

165 AfCFTA Agreement, Article 4.

166 MPP Brochure, cited at note 93 above.

167 Derrick Abudu & Yohannes Ayele, *The AfCFTA and the procurement of pharmaceuticals and vaccines*, ODI Report, October 2024, https://media.odi.org/documents/ODI_Report_-_The_AfCFTA_and_the_procurement_of_pharmaceuticals_and_vaccines.pdf (accessed Feb. 28, 2025).

168 Biovac, *Scaling Up African Vaccine Manufacturing Capacity: Perspectives from the African vaccine manufacturing industry on the challenges and the need for support*, January 2023, Scaling up African vaccine manufacturing capacity | Reports | Wellcome (accessed Feb. 26, 2025).

169 G7ITALIA Report, note 3 above.

170 World Economic Forum, AfCFTA, note 19 above.

171 UNCTAD, note 145 above.

172 G7ITALIA Report, note 3 above.

173 African Union, *Protocol to the Agreement the African Continental Free Trade Area on Investment (AfCFTA Investment Protocol)*, Articles 6–8.

174 Tolulope Anthony Adekola & Bryan Mercurio, note 1 above.

175 MPP TT Agreement Template, Article 6.

176
*Ibid*.

177
*Ibid*.

178 Matther Herder & Ximena Benavides, *Our Project, Your Problem?’ A case study of WHO’s mRNA Technology Transfer Programme in South Africa*, 2023 4(9) *PLOS Global Public Health* 7.

179
*Ibid*. See also, Health Policy Watch, *WHO’s mRNA Vaccine Hub Faces ‘Uncertainty and Structural Weaknesses*, September 30, 2024, https://healthpolicy-watch.news/whos-mrna-vaccine-hub-faces-uncertainty-and-shortfalls/ (accessed Feb. 28, 2025).

180 MPP TT Agreement Template, Article 6. See also Matther Herder and Ximena Benavides, note 178 above, page 11.

181
*Ibid*.

182 See also Tolulope Anthony Adekola & Bryan Mercurio, note 1 above, 13.

183 Tshimanga Kongolo, *TRIPS, The Doha Declaration and Public Health* (2003) 6 Journal of World Intellectual Property 373.

184 World Trade Organization, *Doha Declaration on the TRIPS Agreement and Public Health (Doha Declaration)*, WT/MIN(01)/DEC/2 (November 20, 2001).

185 TRIPS Agreement, Article 31bis. See also WTO, *Amendment of the TRIPS Agreement: Decision of 6 December 2005*, WTO doc WT/L/641, 8 December 2005.

186 Anna S.Y. Wong, Clarke B. Cole & Jillian C. Kohler, *TRIPS Flexibilities and Access to Medicines: An Evaluation of Barriers to Employing Compulsory Licenses for Patented Pharmaceuticals at the WTO* (2022) 168 South Centre Research Paper 28.

187 See also Devika Dutt et al., *An mRNA Technology Transfer Programme and Economic Sustainability in Health Care*, 2024, 102 Bulletin of the World Health Organization 344.

188 AfCFTA Draft IP Protocol, Article 21.

189 AfCFTA Draft IPRs Protocol, Article 23.

190 Reuters, *Moderna to build mRNA vaccine manufacturing facility in Kenya*, March 8, 2022, https://www.reuters.com/business/healthcare-pharmaceuticals/moderna-build-mrna-vaccine-manufacturing-facility-kenya-2022-03-07/ (accessed Feb. 26, 2025).

191 Moderna, *Moderna Finalizes Agreement with the Government of the Republic of Kenya to Establish an mRNA Manufacturing Facility*, March 30, 2023, https://investors.modernatx.com/news/news-details/2023/Moderna-Finalizes-Agreement-with-the-Government-of-the-Republic-of-Kenya-to-Establish-an-mRNA-Manufacturing-Facility/default.aspx (accessed Feb. 26, 2025).

192 Moderna, *Statement on the Kenya Manufacturing Facility*, April 11, 2024. See also Bryan Mercurio, *Moderna Cancels Kenya Vaccine Project due to Familiar Economic Hurdles*, Think Global Health, May 1, 2024.

193
*Ibid*.

194
*Current and Planned Vaccine Manufacturing in Africa*, note 45 above.

195
*Ibid*. See also UNCTAD, note 145 above.

196 Clinton Health Access Initiatives, *A Database of Local Vaccine Manufacturing Commitments and Tech Transfers*, https://www.clintonhealthaccess.org/database/a-database-of-local-vaccine-manufacturing-commitments-and-tech-transfers/ (accessed Feb. 14, 2025).

197 Current and Planned Vaccine Manufacturing in Africa, note 43 above.

198 Partnerships for African Vaccine Manufacturing (PAVM) Framework for Action, March 3, 2022, 14.

199 Health Policy Watch, *Despite Hosting mRNA Hub, South Africa Buys Vaccines from India- Highlighting Tension between Price Pressures and Local Production*, April 20, 2023, https://healthpolicy-watch.news/despite-hosting-mrna-hub-south-africa-buys-vaccines-from-india-highlighting-tension-between-price-and-local-production/ (accessed Feb. 18, 2025).

200 Arianna Martinelli et al., *Collective Licensing Asymmetric Information: The Double Effect of Medicines Patents Pool on Generic Drug Markets*, July 31, 2021, https://papers.ssrn.com/sol3/papers.cfm?abstract_id=3949438 (accessed Feb. 20, 2025).

201
*Ibid*.

202 Lucy Xiaolu Wang, *A Cost Benefit Analysis of the Medicines Patents Pool*, 2023 43 Economics Bulletin 1298–1319.

203 Arianna Martinelli et al., note 200 above.

204 See *Operation Warp Speed: Accelerated COVID-19 Vaccine Development Status and Efforts to Address Manufacturing Challenges*, https://www.gao.gov/products/gao-21-319 accessed on February 28, 2025.

205 Sebastien Morin et al., *The economic and public health impact of intellectual property licensing of medicines for low-income and middle-income countries: a modelling study*, 2022 7(2) The Lancet 169. Purchase guarantees were extensively used by the USA during COVID-19 pandemic.

